# Nitration-induced ubiquitination and degradation control quality of ERK1

**DOI:** 10.1042/BCJ20190240

**Published:** 2019-07-02

**Authors:** Yuanya Zhang, Xiahe Huang, Jinlong Wang, Xiaorong Wang, Xiaofei Liu, Yuhang Chen, Wu Xu, Yingchun Wang

**Affiliations:** 1 State Key Laboratory of Molecular Developmental Biology, Institute of Genetics and Developmental Biology, Chinese Academy of Sciences, No. 1 West Beichen Rd., Beijing 100101, China; 2 University of Chinese Academy of Sciences; 3 Department of Chemistry, University of Louisiana at Lafayette, Lafayette, LA 70504, U.S.A.

**Keywords:** ERK, mass spectrometry, phosphorylation, quality control, tyrosine nitration, ubiquitination

## Abstract

The mitogen-activated protein kinase ERK1/2 (ERKs, extracellular-regulated protein kinases) plays important roles in a wide spectrum of cellular processes and have been implicated in many disease states. The spatiotemporal regulation of ERK activity has been extensively studied. However, scarce information has been available regarding the quality control of the kinases to scavenge malfunctioning ERKs. Using site-specific mutagenesis and mass spectrometry, we found that the disruption of the conserved H-bond between Y210 and E237 of ERK1 through point mutation at or naturally occurring nitration on Y210 initiates a quality control program dependent on chaperon systems and CHIP (C-terminal of Hsp70-interacting protein)-mediated ubiquitination and degradation. The H-bond is also important for the quality control of ERK2, but through a distinct mechanism. These findings clearly demonstrate how malfunctioning ERKs are eliminated when cells are in certain stress conditions or unhealthy states, and could represent a general mechanism for scavenging malfunctioning kinases in stress conditions.

## Introduction

The mitogen-activate protein kinase ERKs (extracellular-regulated protein kinases) play a central role in various cellular processes, including proliferation, migration, apoptosis, and stress response [1]. More than 175 substrates in different cellular compartments can be phosphorylated by ERKs in different scenarios [2]. Thus, the activity of ERKs must be spatiotemporally regulated and the quality of ERKs also must be tightly controlled to achieve optimal function.

In resting cells, ERKs mainly localize in the cytoplasm and anchored by MEKs (mitogen-activated protein kinase kinase) [3], and double phosphorylation of TEY motif at the activation lip by MEKs is necessary for ERK activation. The activated ERKs are then released from MEKs and targeted to different subcellular compartments through binding to scaffold or adaptor proteins. One of the most important subcellular compartments that activated ERKs translocate to is nucleus, where ERKs phosphorylate a plethora of nuclear substrates, including many transcription factors such as Elk [4], to promote gene expression and cell proliferation.

Phosphorylation plays a critical role in regulating ERK activity and localization. It has long been established that TEY motif phosphorylation is important for ERK nuclear translocation [5]. The recent discovery of the nuclear translocation signals (NTS) on ERKs further elucidated the mechanism of ERK nuclear translocation [6,7]. In this model, the Ser-Pro-Ser (SPS) motif in the NTS sequence can be phosphorylated by casein kinase once the canonical TEY motif is phosphorylated by MEKs and then mediates ERK binding to importin 7 and subsequent nuclear translocation [6].

In addition to the TEY and the SPS motifs, ERK activation and subcellular localization could also be affected by post-translational modifications (PTMs) on the other residues. Site-specific mutations of potentially phosphorylatable residues close to the TEY motif revealed that ERK activities were significantly affected in these point mutants, probably due to the abolished ability in accepting modification moieties [8]. For ERK2, G_βγ_-induced autophosphorylation on T188 promotes its nuclear localization to phosphorylate nuclear targets known to cause cardiac hypertrophy [9]. For ERK1, phosphorylation of T198 and T207 has been identified by numerous studies [10], and a recent report showed the detection of Y210 phosphorylation by Western blotting using an in-house made antiserum [11]. Both T198 and T207 mutations were previously shown to reduce ERK1 phosphorylation by MEK by *in vitro* kinase assay [8]. However, it is not known whether these mutations affect ERK1 phosphorylation and localization in living cells. Y210 is close to the _202_TEY_204_ activation motif, Y210F mutation was shown to repress ERK1 activity [11], yet its underlying mechanism still remains elusive. Besides phosphorylation, other types of PTMs occurred on ERKs have been occasionally reported such as ubiquitination, acetylation, and nitration [10]. The regulatory role and the underlying mechanism of these modifications towards ERK activity are largely unknown, though nitration was reported to positively or negatively regulate ERK activity in different scenarios [12,13]. Thus, it is possible and important to discover new PTM sites and novel mechanisms that are critical for the regulation of ERK activity and cellular localization.

Using site-specific mutagenesis, we mutated many potentially phosphorylatable Ser, Thr, and Tyr residues proximal to the TEY motif to their unphosphorylatable counterparts and phosphomimetic residues. We found that Y210F mutation drastically inhibited ERK1 phosphorylation and nuclear translocation. We also found that ERK1 Y210 can be tyrosine nitrated and uncovered a novel tyrosine nitration-induced CHIP-dependent ERK1 degradation pathway, which could serve as an important quality control mechanism for the important kinase when it becomes malfunctioning.

## Materials and methods

### Reagents and antibodies

Peroxynitrite (ONOO^−^), Leptomycin B (LMB), and MG132 were from CalBiochem (Hessen, Germany). Cycloheximide (CHX) was obtained from Sigma (St. Louis, MO). Phosphor-ERK (T202/Y204) antibody, anti-HSP90, and anti-GAPDH were obtained from Cell Signaling Technology (Beverly, MA). Anti-ubiquitin and anti-NitroTyr antibodies were from Millipore (Billerica, MA). Anti-ERK1 antibody was purchased from Santa Cruz Biotechnology (Santa Cruz, CA). Anti-GFP antibody was from Abcam (Cambridge, U.K.). Secondary antibodies including a horseradish peroxidase (HRP)-labeled goat anti-rabbit antibody and a HRP-labeled goat anti-mouse antibody were purchased from CW Biotech (Beijing, China). Protein A Sepharose was from GE Healthcare Bioscience (Sweden). DAPI and ProLong Gold antifade reagent were from Invitrogen (Waltham, MA).

### Cell culture and transfections

HEK293T cells were cultured in high-glucose Dulbecco's modified Eagle's medium (DMEM, HyClone, Logan, UT) supplemented with 10% fetal bovine serum (FBS), 1 mM sodium pyruvate, 100 units/ml penicillin and 100 µg/ml streptomycin (GIBCO, CA) at 37°C with 5% CO_2_. For transfection, TurboFect *in vitro* Tansfection Reagent (Thermo Fisher Scientific, Inc., Lithuania) and Lipofectamine 2000 (Invitrogen, CA) were used for transfecting plasmids and siRNA, respectively.

### Site-directed mutagenesis

All site-directed mutagenesis was performed using the QuikChange II XL site-directed mutagenesis system (Stratagene, La Jolla, CA). The mutations were then confirmed by DNA sequencing. In total, 19 site-specific mutants were generated for the current study (Supplementary Table S1).

### Immunofluorescence

Transfected 293T cells were plated on coverslips with ∼50–60% confluence and allowed to attach and spread overnight in the starvation medium. The cells were then either treated with 10% FBS for 10 min to activate ERKs or untreated. After the treatment, the cells were washed with ice-cold PBS, fixed with 4% paraformaldehyde for 15 min, permeabilized with 0.1% Triton X-100 for 10 min, and then blocked with 10% BSA in PBS containing DAPI in a closed chamber at 37°C for 1 h. The coverslips were subsequently washed, mounted on glass slides using ProLong Gold antifade reagent, and visualized by Leica fluorescence microscopy (DMI6000B, Germany).

### *In vitro* nitration assay

Ectopically expressed GST-ERK1 was pulled down from the WCL of transfected HEK293T cells by GST.Bind Resin (Novagen, San Diego, CA) at 4°C for 2 h. The beads bound with GST-ERK1 were then incubated with the freshly collected HEK293T cell lysate. Nitration of GST-ERK1 was stimulated by adding 500 µM ONOO^−^ to the reaction system, and the reaction was allowed to proceed at 37°C for 30 min [14]. After the reaction, the beads were washed with ice-cold RM buffer to remove non-specific binding proteins. The GST-ERK1 on the beads was released either by boiling with 3× SDS sample buffer for Western blotting or with 1.5 M urea in 25 mM NH_4_HCO_3_ (pH 7.4) for mass spectrometry (MS) analysis.

## Results

### Site-specific mutagenesis of potential ERK1 phosphorylation sites

The three highly conserved and potentially phosphorylatable residues T198, T207, and Y210 were chosen for site-specific mutagenesis (Supplementary Figure S1A,B), which replaces the targeted residues with structurally similar but unphosphorylatable ones. Thus, we obtained T198A, T207A, and Y210F mutants in which Ser or Thr residues were replaced with Ala and Tyr was replaced with Phe [8]. Since neither T198A nor T207A significantly affects ERK1 nuclear translocation in response to mitogenic stimulation (Supplementary Figure S2), we focused our functional investigation hereafter only on Y210. Suppression of TEY motif phosphorylation by Y210F mutation indicates that this mutation could lead to loss of ERK1 function (Supplementary Figure S1B), which may significantly affect a plethora of ERK-dependent cellular functions.

### Mutation of Y210 inhibits ERK1 phosphorylation and nuclear translocation

**Figure 1.**
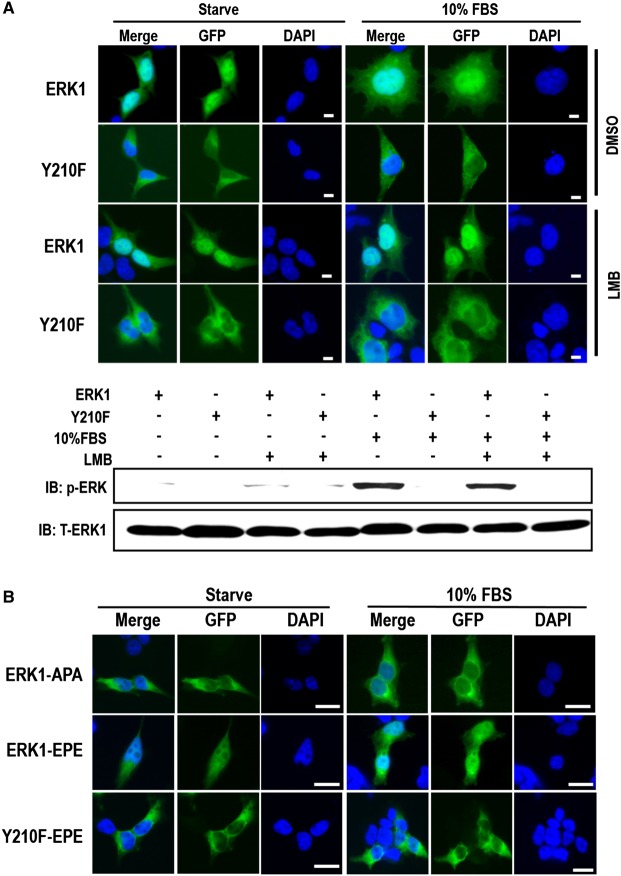
Y210F mutation abrogates ERK1 nuclear localization. (**A**) HEK293T cells expressing GFP-ERK1 (ERK1) or GFP-ERK1-Y210F (Y210F) were serum-starved overnight and then treated with the nuclear export inhibitor LMB (40 mM) or vehicle (DMSO) for 3 h as indicated. The cells were then stimulated with 10% FBS or unstimulated (Starve) for 10 min as indicated. The cells were then fixed, stained with DAPI, and imaged with fluorescence microscopy (upper panel). Bar = 10 µm. The ERK phosphorylation level (TEY motif phosphorylation) in each treatment was detected with Western blotting, and total ectopically expressed ERK1 was also probed as the loading control (lower panel). Shown is the representative result of three biological replicates. (**B**) HEK293T cells expressing phosphomimetic mutant of the ERK1 nuclear translocation signal (NTS) (ERK1-EPE), the unphosphorylable mutant of NTS (ERK1-APA), and the triple-mutant Y210F-EPE were serum-starved and then stimulated with 10% FBS for 10 min or unstimulated (Starve). The cells were then fixed, stained with DAPI, and imaged with fluorescence microscopy. Bar = 25 µm.

To answer whether Y210F mutation affects ERK1 nuclear translocation, 293T cells and HeLa cells expressing N-terminal GFP fusion of ERK1 or Y210F were examined for nuclear localization. While ERK1 was still in nucleus in response to 10% FBS stimulation as expected, Y210F was almost exclusively retained in the cytoplasm in both serum-starved and FBS-stimulated cells (Figure 1A and Supplementary Figure S3). To exclude the possibility that Y210F may be able to translocate into the nucleus and rapidly exported from the nucleus in a nuclear export signal (NES)-dependent active transport manner facilitated by NES-containing MEKs [15], we treated the cells with LMB, a drug that specifically inhibits NES-dependent nuclear exportation [15]. The LMB treatment did not result in observable changes of TEY motif phosphorylation of both ERK1 and Y210F (Figure 1A) and induced significant nuclear accumulation of ERK1 in quiescent cells as expected. However, LMB treatment did not induce any observable Y210F accumulation in nuclei in both starved and serum-stimulated cells (Figure 1A). Therefore, the inhibition of Y210F nuclear localization is due to its defect in nuclear import, but not faster-than-normal nuclear export.

Activated ERK1 nuclear translocation requires double phosphorylation of the two Ser residues on the consensus SPS motif [6,7]. Y210F mutation may prevent the SPS phosphorylation and hence inhibit ERK1 nuclear translocation. To test this possibility, we generated the phosphomimetic mutants and the unphosphorylatable counterparts of the SPS motif for both ERK1 and Y210F as previously described [6], in which the two Ser residues of the SPS motif were replaced with Glu (EPE) and Ala (APA), respectively. While EPE mutation induced constitutive ERK1 nuclear localization in both serum-starved and FBS-stimulated cells, which is consistent with previous reports [6,7], no nuclear localization was observed for Y210F-EPE (Figure 1B), suggesting that the activated NTS is not sufficient to drive Y210F nuclear localization. As a control, APA mutation completely abolished ERK1 nuclear localization in both quiescent and serum-stimulated cells, which is also consistent with previous reports [6,7].

To investigate if the observed phenotype of Y210F is owing to the loss of phosphorylation potential of the Y210 residue, we generated three other site-specific mutants Y210E, Y210S, and Y210T. Y210E is a possible phosphomimetic mutation, and Y210S and Y210T could retain the phosphorylation potential at this particular residue. Again, all mutations significantly inhibited ERK1 TEY motif phosphorylation and completely abolished ERK1 nuclear translocation in both quiescent and serum-stimulated cells (Supplementary Figure S4). Apparently, it is not the lost potential of phosphorylation *per se* but the unique structure of the bulky side chain of the Y210 residue that is critical for the optimal ERK1 activation and localization.

### The H-bond between Y210 and E237 is critical for ERK1 activation and nuclear localization

**Figure 2.**
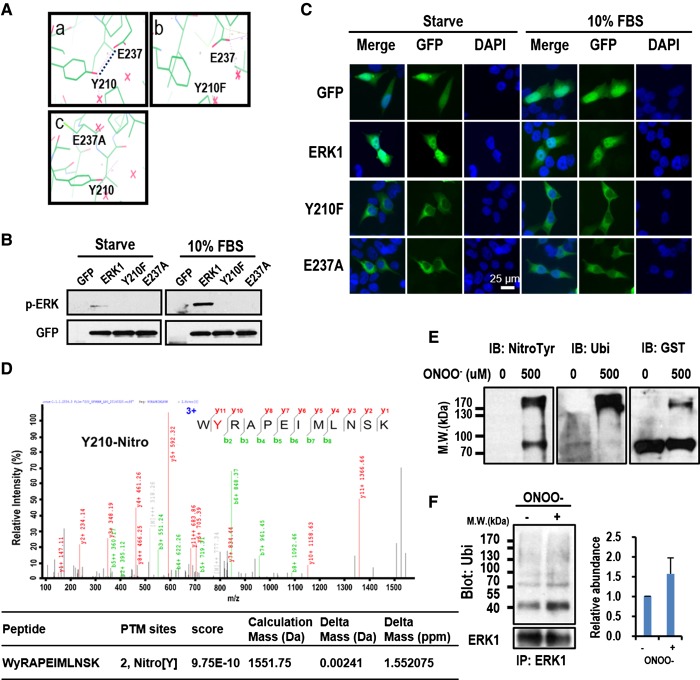
The H-bond between Y210 and E237 is critical for ERK1 phosphorylation and nuclear localization. (**A**) A part of ERK1 crystal structure (PDB accession: 2ZOQ) showing an H-bond between Y210 and E237 (panel a, dashed line). Simulations of the structures for Y210F and E237A mutants show that the H-bond between Y210 and E237 in wild-type ERK1 is disrupted in the mutants (panels b,c). (**B**) HEK293T cells expressing GFP, ERK1, Y210F, or E237A were serum-starved and stimulated with 10% FBS for 10 min or unstimulated (Starve), WCL were immunoblotted for phosphor-ERK and total ERK, respectively. Shown is the representative result of three biological replicates. (**C**) Serum-starved cells described in (**B**) were stimulated with 10% FBS or unstimulated (Starve). The cells were then fixed, stained with DAPI, and imaged with fluorescence microscopy. (**D**) The mass spectrum shows the identification of Y210 nitration. HEK293 cells expressing GFP-ERK1 were serum-starved overnight and then stimulated with 10% FBS for 10 min, GFP-ERK1 was then immunoprecipitated from the harvested cell lysates and analyzed wtih LC–MS/MS. The residue in red in the peptide sequence indicates the tyrosine nitration site (upper panel). The spectrum was annotated with the software pFind, and the score and mass accuracy were also shown (bottom panel). (**E**) *In vitro* tyrosine nitration assay of ERK1. GST-ERK1 was pulled down from WCL and then incubated with freshly collected WCL of 293T cells in the presence or absence of 500 mM peroxynitrite. The fusion protein was then separated with SDS–PAGE and probed for nitrotyrosine, ubiquitination, and GST using respective specific antibodies. The total level of GST-ERK1 was also probed as the loading control using anti-GST antibody. This experiment was performed in triplicates and only the representative result was shown. (**F**) Endogenous ERK1 was IPed from serum-starved HEK293T cells treated with 50 mM peroxynitrite for 4 h. ERK1 ubiquitination was detected by Western blotting and the results were quantified from triplicated experiments (mean ± s.d.).

To answer why the Y210 residue of ERK1 is so critical for maintaining optimal ERK1 activity and nuclear localization, the resolved structure of ERK1 was downloaded from Protein Data Bank (PDB) and analyzed with the software Wincoot. We found that there is an H-bond between the phenolic hydroxyl group of Y210 and the carboxyl group on the side chain of E237 (Figure 2A). Structure simulation using the Wincoot software revealed that mutations on the two residues, including Y210F and E237A, can disrupt the H-bond (Figure 2A(b,c)). To further confirm the simulation and the functional significance of the H-bond, E237 was also mutated to Ala. As expected, E237A mutations significantly inhibit TEY motif phosphorylation and abolish ERK1 nuclear translocation in response to serum stimulation (Figure 2B,C and Supplementary Figures S5 and S6), which phenocopy Y210F mutation. Taken together, Y210 and E237 form an H-bond that is important for optimal ERK1 function.

In addition to the mutations, PTMs conjugated on Y210 may also disrupt the H-bond due to steric restriction or the change of physiochemical properties [16]. Phosphorylation and nitration are two major PTMs that are frequently occurred on tyrosine residues. Thus, we tried to identify potential PTMs on Y210 using MS. Intriguingly, we successfully identified Y210 nitration from serum-stimulated or over-passaged cells (Figure 2D), but failed to identify Y210 phosphorylation in any culturing conditions we tried. The tyrosine nitration of ERK1 induced by peroxynitrite, an oxidant-producing tyrosine nitration [17], was also confirmed by MS (Supplementary Figure S7) and Western blotting using specific anti-nitrotyrosine and anti-ubiquitination (anti-ubi) antibodies (Figure 2E) [18]. Significantly, we observed a significant fraction of tyrosine-nitrated ERK1 with up-shifted molecular mass (>170 kDa) (Figure 2E), which is indicative of ubiquitination. Indeed, when probed with anti-ubiquitination antibody, strong ubiquitination signals were detected. Increased ubiquitination of endogenous ERK1 was also observed upon ONOO^−^ treatment (Figure 2F). This observation suggests that tyrosine nitration of ERK1 in response to nitroxidative stress can induce ERK1 ubiquitination.

One of the consequences of the tyrosine nitration is the significant decrease in the p*K*_a_ of the –OH group from 10–10.3 to 7.2–7.5 [19]. Thus, it is safe to deduce that the phenolic –OH group of almost all ERK1 Y210 are protonated under physiological conditions, but will drastically deprotonated to form phenolate due to the drop of p*K*_a_ when nitration occurs on this residue [16]. The phenolate is not able to form the H-bond with E237 due to the loss of the proton. Together, nitration of Y210 may represent a novel mechanism by which ERK1 activity is regulated through the disruption of the H-bond in certain conditions such as in aged cells or cells under oxidative stress.

### Y210f mutation promotes Hsp90 binding to ERK1 and inhibits ERK1 nuclear localization

**Figure 3.**
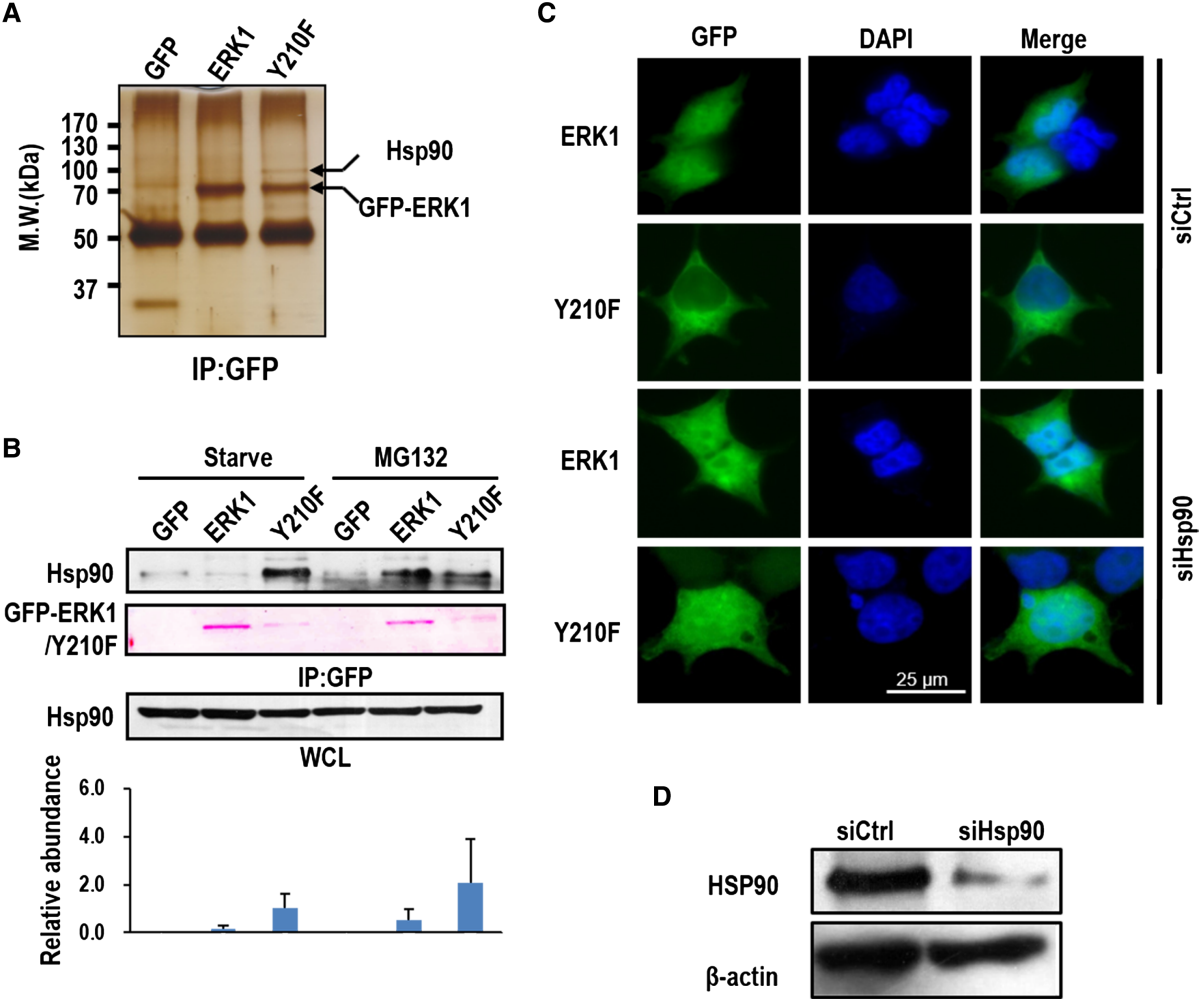
ERK1 Y210F mutation promotes the recruitment of Hsp90. (**A**) Ectopically expressed GFP-ERK1 (ERK1) or GFP-ERK1 Y210 (Y210) in 293T cells were IPed from the WCL with anti-GFP antibody and silver-stained after separation with SDS–PAGE. The protein bands indicated by the arrows were excised from the gel and analyzed with MS. (**B**) GFP fusion of ERK1 or Y210F in serum-starved or MG132-treated (5 µM) HEK293 cells was IPed with anti-GFP antibody and probed for Hsp90 by Western blotting. Total levels of Hsp90 in the WCL were also probed by Western blotting as the loading control. The bars indicate the quantified Hsp90 level from three IP replicates (mean ± s.d.). The bands in red were stained by Ponceau in this and all the other figures. (**C**) 293T cells expressing the GFP fusion of ERK1 or Y210F were transfected with siRNA targeting for Hsp90 (siHsp90) or non-targeting siRNA (siCtrl). The cells were fixed 72 h after the transfection, then stained with DAPI, and imaged with fluorescence microscopy. (**D**) The knockdown efficiency of Hsp90 was detected by Western blotting using anti-Hsp90 antibody. β-Actin was also probed as the loading control. Shown is the representative result of three biological replicates.

To examine if the H-bond disruption influences the binding of ERK1-interacting proteins critical for the regulation of its activity and localization, ERK1 and Y210F were immunoprecipitated (IPed) from the whole cell lysates (WCL), and the co-IPed proteins were separated by SDS–PAGE and visualized with silver staining. We found a protein, which was subsequently identified as the heat shock protein 90 (Hsp90) by MS (Figure 3A), specifically co-IPed with Y210F. The specific binding of Hsp90 to Y210F was further confirmed by Western blotting (Figure 3B). Interestingly, in cells treated with MG132, the potent and specific inhibitor of 26S proteasome, a significant amount of Hsp90 was also observed to bind to ERK1 (Figure 3B). The subpopulation of Hsp90-bound ERK1 species is presumably the consequence of abnormal folding, either by spontaneous mutation or by PTM that disrupts the aforementioned H-bond.

Binding of Hsp90 could have a causal effect on the abolished Y210F nuclear localization due to size increase in forming the complex. Indeed, in cells partially depleted of Hsp90 by siRNA, Y210F regained the ability for nuclear localization (Figure 3C,D). Thus, Hsp90 binding could serve as an important mechanism to prevent the nuclear localization of malfunctioning ERK1.

### Y210f mutation induces ERK1 ubiquitination and degradation

**Figure 4.**
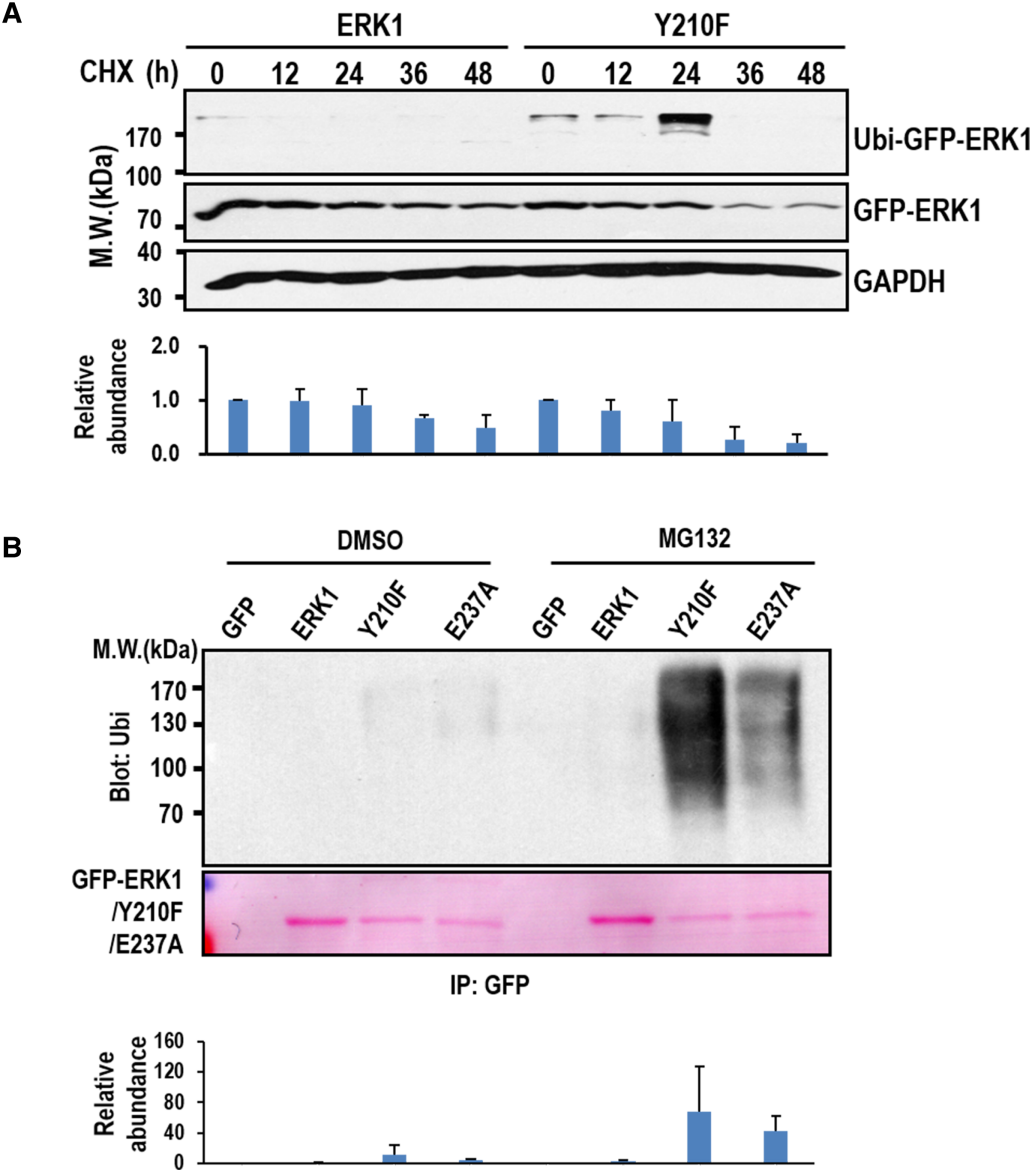
Y210F mutation promotes ERK1 ubiquitination and degradation. 293T cells expressing GFP-ERK1 (ERK1) or GFP-Y210F (Y210F) were treated with CHX for the indicated times. (**A**) The expression level of the fusion proteins was probed by Western blotting using anti-GFP antibody and quantified from triplicated experiments (mean ± s.d.). The up-shifted bands are probably the ubiquitinated ERK1 or Y210F. (**B**) HEK293T cells expressing GFP, GFP-ERK1 (ERK1), GFP-Y210F (Y210F), or GFP-E237A (E237A) were treated with MG132 or vehicle (DMSO) for 4 h and then lysed. GFP and the other GFP fusion proteins were IPed from the WCL and probed for ubiquitination by Western blotting using anti-ubi antibody. Quantification of ubiquitination was performed using results from triplicated experiments (mean ± s.d.).

Binding of Hsp90 strongly suggests that Y210F mutant is abnormally folded with two possible and opposing consequences, i.e. refolding and degradation, and both could be mediated by Hsp90 as observed for other client proteins [20–22]. Because Y210F mutation is unstable and the resulted conformational change might not be reversible, degradation is more likely to occur than refolding. To confirm this, we examined the degradation of ERK1 and Y210F using cells expressing GFP fusion of ERK1 or Y210F. As expected, the levels of both Y210F and ERK1 were continuously decreasing during the time course of CHX treatment, which inhibits protein translation (Figure 4A). However, the decreasing of Y210F is much faster than that of ERK1. At the time point 48 h, only trace amount of Y210F was detected, whereas the amount of ERK1 was much higher, suggesting that Y210F is less stable and more susceptible to degradation. Significantly, the molecular mass of a portion of Y210F was up-shifted (>170 kDa), which is indicative of ubiquitination. The up-shifted Y210F accumulated to a maximal level at the time point 24 h, and then disappeared after 36 h, presumably being completely degraded by the proteasome. In support of this, ubiquitination can be detected for both GFP-ERK1 and GFP-Y210F IPed from 293T cells by Western blotting, and MG132 treatment induced significantly higher accumulation of ubiquitination on Y210F than on ERK1 (Figure 4B), indicating that Y210F is more liable to ubiquitination and subsequent degradation. Indeed, much less Y210F can be IPed from the same amount of WCL compared with ERK1 (Figure 4B), and much weaker fluorescence has been consistently observed in cells expressing GFP-Y210F than in cells expressing GFP-ERK1. Both observations further support that the degradation of Y210F is much faster than that of ERK1. Remarkably, the E237A mutant is also highly ubiquitinated, though the level is lower than that of the Y210F (Figure 4B). This provides additional evidence that disruption of the H-bond leads to ERK1 ubiquitination and degradation.

### CHIP is the major E3 ligase that mediates ubiquitination and degradation of abnormally folded ERK1 with disrupted H-bond between Y210 and E237

**Figure 5.**
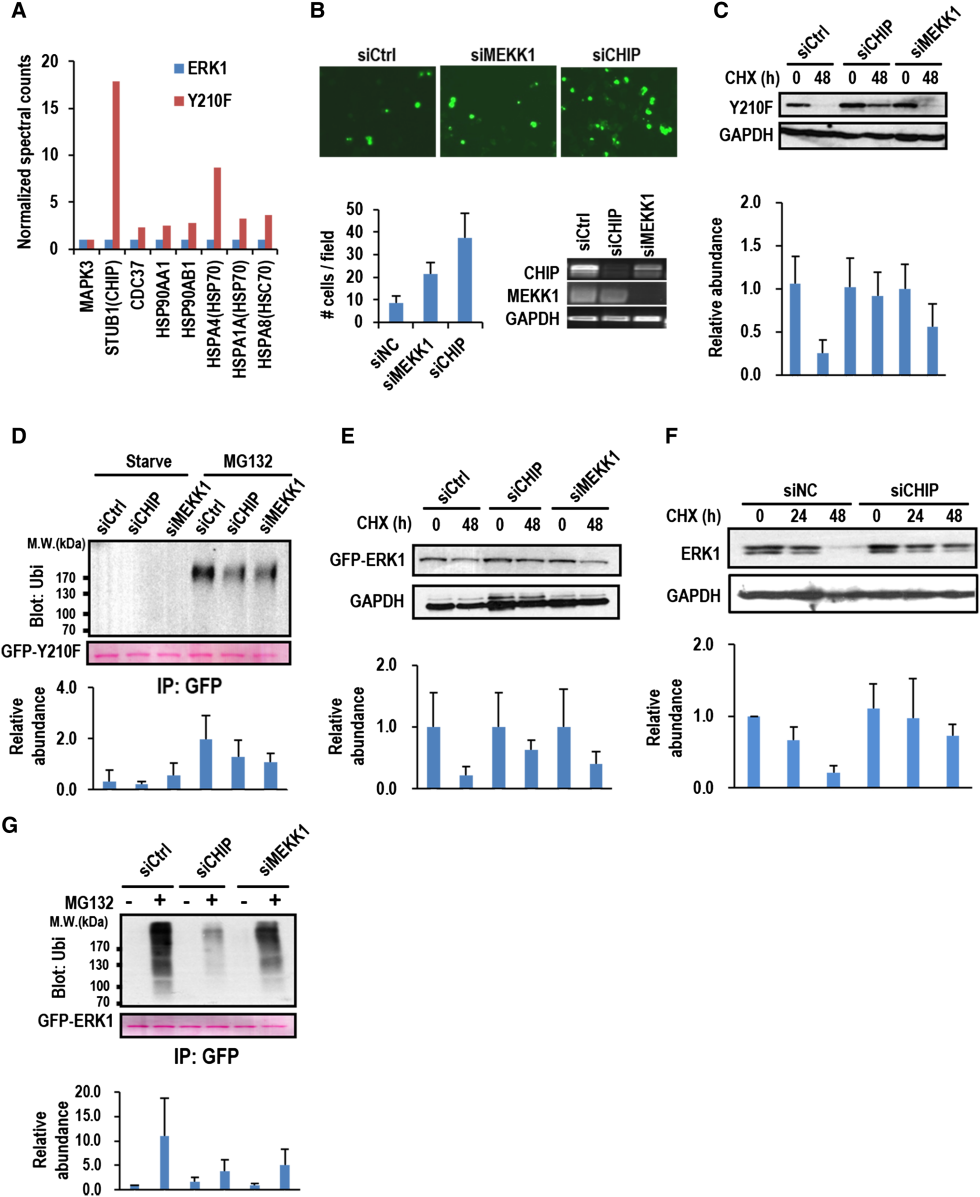
CHIP is the major E3 ubiquitin ligase mediating Y210F ubiquitination and degradation. (**A**) GFP-ERK1 (ERK1) or GFP-Y210F (Y210F) was IPed from 293T cells. The co-IPed proteins were identified by MS, and the spectra count of each protein co-IPed with GFP-ERK1 was used as the standard for normalization. The bars indicate the fold changes of the spectra counts of the identified proteins over the standards. (**B**) 293T cells expressing GFP-Y210F were transfected with siRNA targeting for MEKK1 (siMEKK1) or CHIP (siCHIP) or non-targeting siRNA (siCtrl) as indicated. The cells were imaged with fluorescence microscopy, and the numbers of cells with fluorescent aggregates were counted with Image J. The bars indicate the numbers of cells with fluorescence aggregates per field (mean ± s.d.). The knockdown efficiency of MEKK1 and CHIP was detected by RT-PCR. (**C**) The same transfected cells in (**B**) were treated with CHX for the indicated times and then lysed. The WCL was probed for Y210F by Western blotting using anti-GFP antibody. GAPDH was probed as the loading control (upper panel). The bars show the quantitative results from three independent experiments (mean ± s.d.) and were aligned with the corresponding lane in the upper panel (lower panel). (**D**) The same transfected cells in (**B**) were treated with MG132 or untreated (Starve) for 4 h and then harvested. GFP-Y210F was IPed from the WCL and probed for ubiquitination by Western blotting using anti-ubi antibody (upper panel). The bars in the lower panel show the quantified results of Y210F ubiquitination from three independent experiments (mean ± s.d.) and were aligned to the corresponding lanes in the upper panel. (**E** and **G**) The results from the same experiments described in (**C**) and (**D**), respectively, except that the cells used were expressing GFP-ERK1 instead of GFP-Y210F. (**F**) HEK293T cells were transfected with siRNA targeting for CHIP (siCHIP) or non-targeting siRNA (siCtrl) as indicated. The cells were treated with CHX for 24 h, 48 h, or untreated (0 h). The total level of endogenous ERK1 was detected by Western blotting using anti-ERK1 antibody. GAPDH was probed as the loading control. Quantification was performed using results from triplicated experiments (mean ± s.d.).

To find proteins that potentially facilitate Y210F degradation, total proteins co-IPed with ERK1 and Y210F were identified by LC–MS. The quantitative analysis based on the normalized peptide spectral counts revealed that substantially more amount of Hsp90, Hsp70, Hsc70, Cdc37, and CHIP were identified to interact with Y210 than with ERK1 (Figure 5A). Hsp90 and Cdc37 form a chaperon complex that regulates folding and maturation of kinases [23–25], whereas binding of Hsp70 and Hsc70 recruits the E3 ubiquitin ligase CHIP for the ubiquitination and degradation of kinases [26,27]. The preferential binding of CHIP determines the fate of Y210F, i.e. ubiquitination and degradation. Indeed, in CHIP-depleted 293T cells, many more fluorescent aggregates formed by ectopically expressing GFP-Y210F were observed (Figure 5B and Supplementary Figure S8). The aggregated GFP-Y210F could otherwise be scavenged through CHIP-mediated ubiquitination and degradation as shown in the control cells (Figure 5B and Supplementary Figure S8). Other than CHIP, MEKK1 (mitogen-activated protein kinase kinase kinase 1) has previously been shown as the E3 ubiquitin ligase-mediating ERK ubiquitination and degradation in cells under osmotic stress [28]. Depletion of MEKK1 in 293T cells expressing GFP-Y210F also increased the number of cells with fluorescent aggregates, but to a lesser extent (Figure 5B). This observation suggests that Y210F degradation is more repressed in CHIP-depleted cells than in MEKK1-depleted cells, and this was further confirmed by the Western blotting for the detection of the Y210F levels in CHIP- and MEKK1-depleted cells treated with CHX for 48 h (Figure 5C). Intriguingly, only marginal difference can be observed between the ubiquitination level of Y210F in CHIP-depleted cells and in MEKK1-depleted cells, though both are significantly lower than that in the control cells (Figure 5D). Taken together, the results suggest that both CHIP and MEKK1 can mediate ubiquitination of Y210F. However, CHIP, but not MEKK1, takes the major role in mediating the degradation of Y210F.

To further answer whether CHIP is also the major E3 ubiquitin ligase for ERK1, 293T cells depleted of CHIP or MEKK1 were treated with CHX to inhibit protein synthesis and examined for the stability of GFP-ERK1. In contrast with GFP-Y210F, GFP-ERK1 degradation can be significantly inhibited by depletion of either E3 ligase, though the extent of inhibition is slightly greater in the CHIP-depleted cells (Figure 5E). In addition, the degradation of endogenous ERK1 is also significantly inhibited by the depletion of CHIP (Figure 5F). Interestingly, the depletion of CHIP significantly reduced ERK1 ubiquitination, whereas the depletion of MEKK1 only moderately reduced ERK1 ubiquitination (Figure 5G). Such inconsistency is presumably owing to the different sites ubiquitinated by CHIP and MEKK1, which might have different efficiency in mediating ERK1 degradation.

### K294 and K317 ubiquitination targets ERK1 for degradation

**Figure 6.**
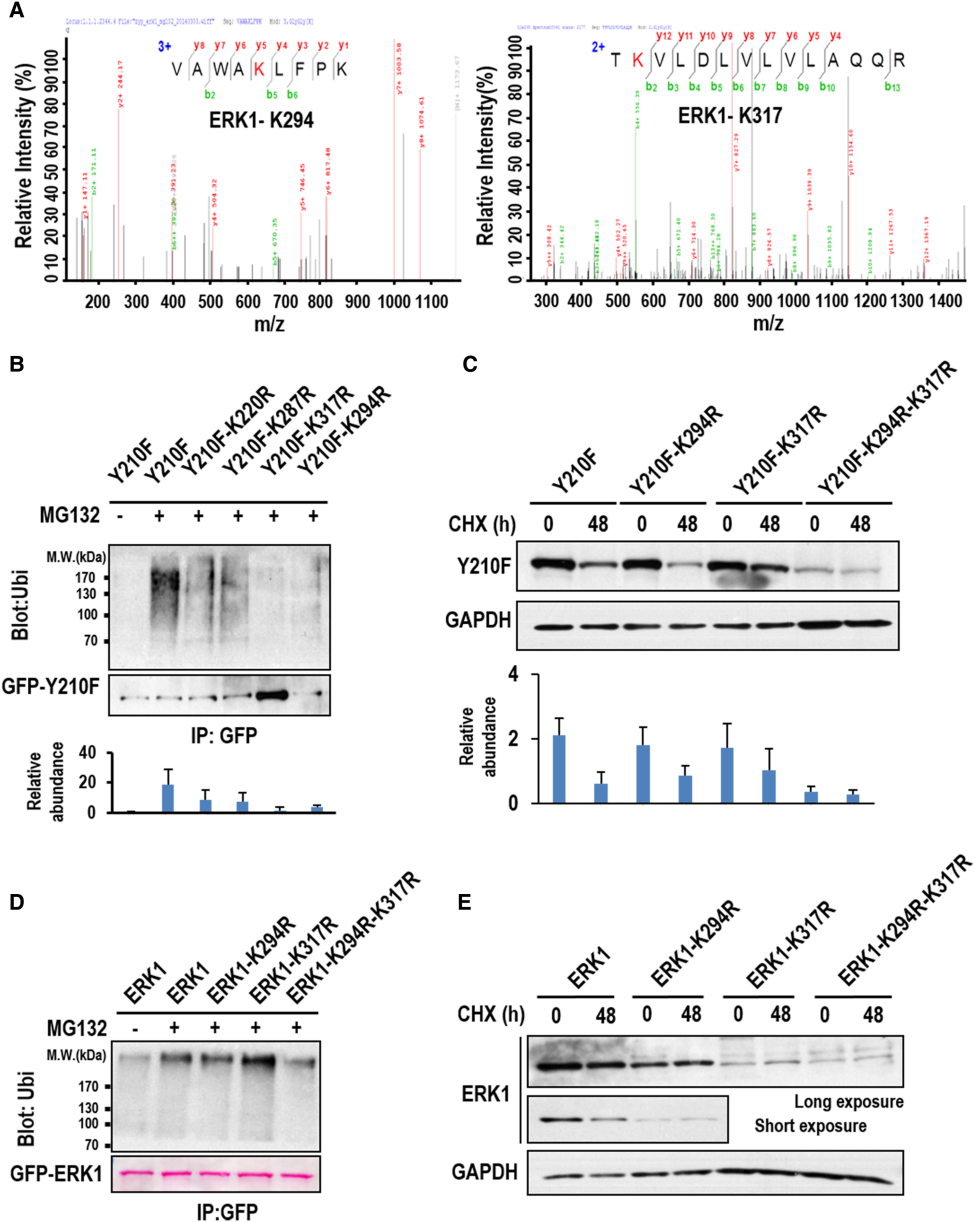
K294 and K317 ubiquitination mediates ERK1 degradation. (**A**) The mass spectra show the identification of the two peptides from ERK1 ubiquitinated at K294 and K317, respectively. The residues in red in the peptide sequences indicate ubiquitination sites. (**B**) 293T cells expressing site-specific mutants generated from Y210F were treated with MG132 for 4 h or untreated as indicated. The cells were then lysed and the GFP fusion proteins were IPed using anti-GFP antibody before probed for ubiquitination by Western blotting using anti-ubi antibody. (**C**) 293T cells expressing GFP fusion of different site-specific mutants generated from Y210F were treated with CHX for 48 h or untreated (0 h). The total level of the fusion proteins was detected by Western blotting using anti-GFP antibody. GAPDH was probed as the loading control. (**D** and **E**) 293T cells expressing site-specific mutants generated from ERK1 as indicated were treated and assayed as described in (**B**) and (**C**), respectively. Quantifications for Western blot results, when applicable, were performed from three independent experiments (mean ± s.d.).

After establishing CHIP as the major E3 ubiquitin ligase for the degradation of a subpopulation of abnormally folded ERK1 represented by Y210F, it is interesting to know which sites on ERK1 are ubiquitinated and important for ERK1 degradation. Using MS, many ubiquitination sites were identified from Y210F including K220, K287, K294, and K317 (Figure 6A and Supplementary Figure S9). To determine which Lys sites are important for Y210F degradation, site-specific mutants were generated on Y210F each with a specific Lys residue replaced by Arg. The mutants were allowed to express in 293T cells, which were subsequently treated with MG132 and then IPed and probed for the ubiquitination level by Western blotting. The result shows that neither K220R nor K287R mutation reduces Y210F ubiquitination in MG132-treated cells, whereas both K294R and K317R mutations significantly reduce Y210F ubiquitination (Figure 6B). Intriguingly, the degradation was significantly inhibited for the double-mutant Y210F-K317R and the triple-mutant Y210F-K294R-K317R compared with that of Y210F, whereas the degradation of Y210F-K294R is not inhibited (Figure 6C). This observation suggests that ubiquitination of K317, but not K294, is critical for Y210F degradation.

To investigate if the two ubiquitination sites are also important for the degradation of ERK1, site-specific mutants ERK1-K294R, ERK1-K317R, and ERK1-K294R-K317R were examined for the ubiquitination level. The result shows that the double mutations, but not the single mutation, significantly reduce ubiquitination of ERK1 (Figure 6D). Nevertheless, the mutation of either single site or both can significantly inhibit ERK1 degradation (Figure 6E), indicating that ubiquitination of both sites is necessary for effective ERK1 degradation. It is worth noting that the total levels of ERK1-K317R and ERK1-K294R-K317R before CHX treatment are much lower than those of ERK1 and ERK1-K294R (Figure 6E), which is probably due to the inhibition of translation through an uncharacterized mechanism caused by the mutations. Altogether, these observations suggest that ERK1 requires ubiquitination of both K294 and k317 for efficient degradation, whereas ubiquitination of K317 alone is sufficient for Y210 degradation.

### Disruption of the H-bond between Y193 and E220 induces ERK2 ubiquitination and degradation independent of HSP90 and CHIP

**Figure 7.**
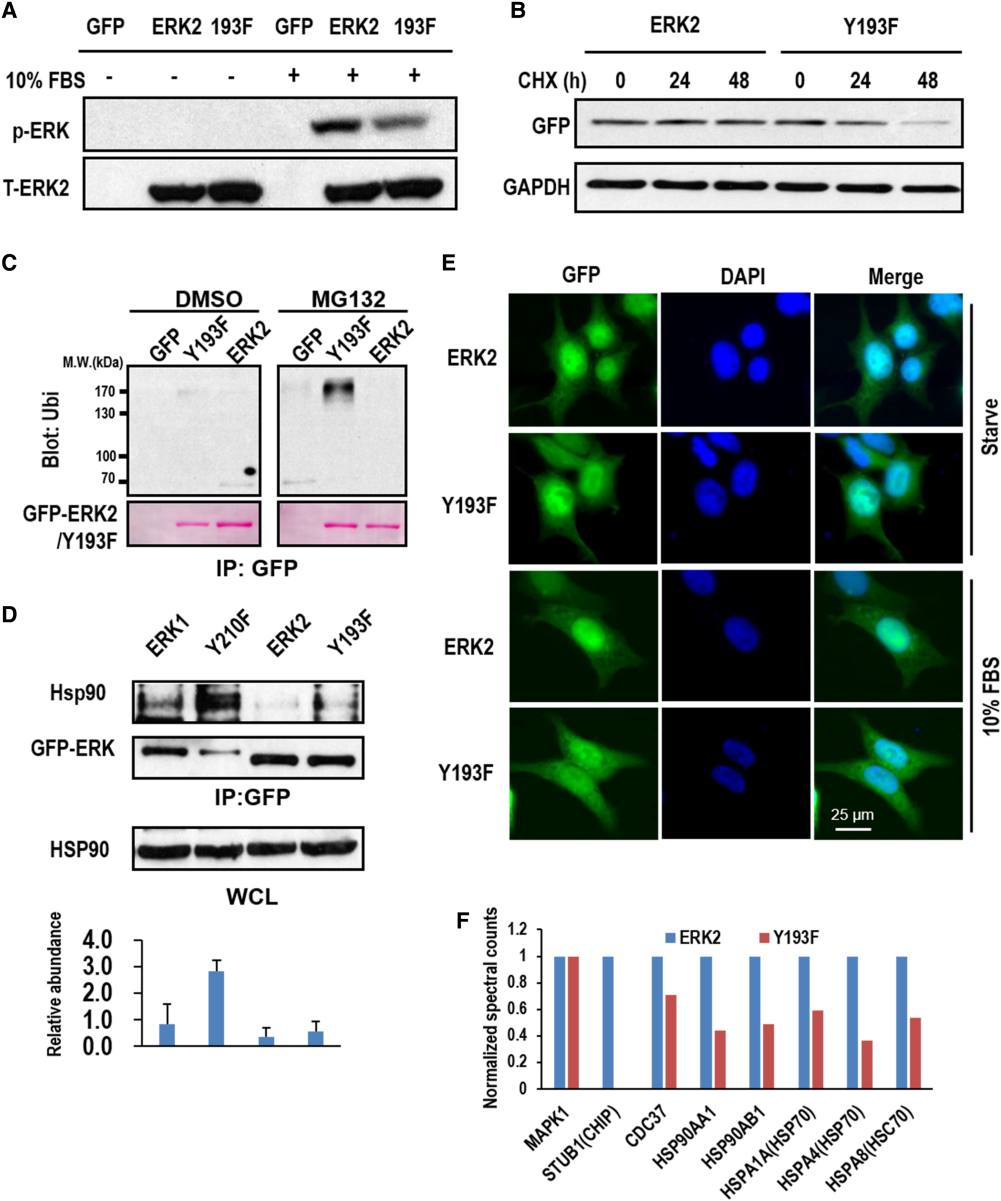
Y193F mutation promotes ERK2 ubiquitination and degradation but via a distinct mechanism from that of ERK1. (**A**) Western blotting detection of the total and the phosphorylation levels of GFP fused ERK2 or Y193F in transfected 293T cells treated with 10% FBS or untreated as indicated. The cells expressing GFP was used as the control. (**B**) Western blotting detection of the expression levels of GFP-ERK2 and GFP-Y193F in transfected 293T cells treated with CHX for the indicated times. GAPDH was also probed for the loading control. (**C**) 293T cells expressing GFP, GFP-ERK2, and GFP-Y193F were treated with MG132 or vehicle (DMSO). The ectopically expressed proteins were IPed with anti-GFP antibody and probed for ubiquitination by Western blotting using anti-Ubi antibody. (**D**) GFP fusions of indicated proteins or their point mutants were IPed from the corresponding transfected 293T cells, and co-IPed Hsp90 was probed by Western blotting. Total Hsp90 in the transfected cells was also probed as the loading control. The results were quantified from triplicated experiments (mean ± s.d.). (**E**) Fluorescence images show the subcellular localization of the GFP fusions of ERK2 and its point mutants in transfected 293T cells. The cells were either treated with 10% FBS or starved as indicated. (**F**) GFP-ERK2 (ERK2) or GFP-Y210F (Y193F) was IPed from 293T cells. The co-IPed proteins were identified by MS, and the spectra count of each protein co-IPed with GFP-ERK2 was used as the standard for normalization. The bars indicate the fold changes in the spectra counts of the identified proteins over the standards.

To investigate if ERK2, the isoform of ERK1, follows the same mechanism to degrade as ERK1 does, site-specific mutations were also generated on ERK2 Y193 and E220, the two residues homologous to ERK1 Y210 and E237, respectively, and forming an H-bond as well. The N-terminal GFP fusions of ERK2 and Y193F were expressed in HEK293 cells. Indeed, the disruption of the H-bond by the Y193F mutation significantly represses ERK2 TEY phosphorylation and promotes ERK2 ubiquitination and degradation (Figure 7A–C), confirming the significance of the H-bond in maintaining the stability and function of ERK2. However, the inhibition of TEY phosphorylation is only partial, and a significant level of phosphorylation can still be reproducibly detected for ERK2 Y193F (Figure 7A), which is contrast with the nearly complete inhibition of phosphorylation of ERK1 Y210F (Supplementary Figure S1B). Moreover, the Y193F mutation does not promote HSP90 binding, though the low level of Hsp90 can be detected to bind to both ERK2 and the Y193F (Figure 7D). Consequently, the nuclear translocation of ERK2 Y193F is not abolished (Figure 7E), which is in sharp contrast with ERK1 Y210F. Taken together, these observations suggest that the H-bond between Y193 and E220 is also important in the regulation of ERK2 stability and function, but the degradation of Y193F may follow a distinct pathway independent of HSP90 and CHIP, the latter was not identified as an ERK2 Y193F-interacting protein by Co-IP and MS (Figure 7F).

## Discussion

**Figure 8.**
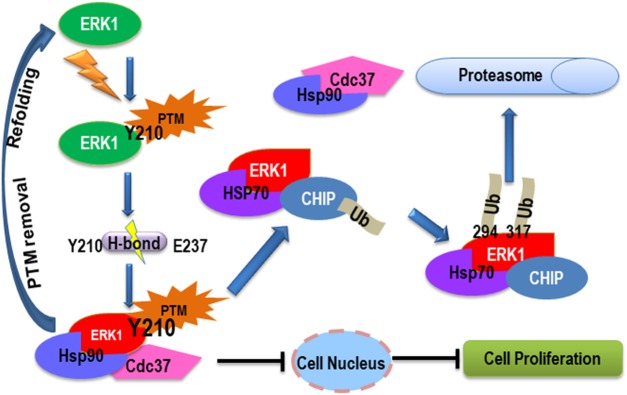
The diagramed model shows Y210 modification-induced ERK1 ubiquitination and degradation. Once Y210 is modified, the H-bond between Y210 and E237 is disrupted resulting in aberrant ERK1 folding and subsequent recruitment of Hsp90. Binding of Hsp90 can facilitate either the refolding and degradation of the misfolded ERK1, depending on whether the niche where ERK1 resides favor refolding or degradation. If the PTM conjugated on Y210 can be removed and the H-bond can be reformed, the refolding attempt could result in renatured proteins with full function. However, if the disrupted H-bond cannot be re-connected due to the failure of removing the PTM on Y210, Hsp90 will facilitate the recruitment of the other proteins such as the Hsp70–CHIP complex, and the latter can ubiquitinate and target ERK1 for degradation through the 26S proteasome system. The binding of Hsp90 also inhibits ERK1 nuclear entry, a secondary effect.

Spatiotemporal regulation of ERK activity has been extensively studied. However, little information regarding the quality control of ERKs has been available. Here, we described a nitration-induced and CHIP-dependent quality control mechanism for ERK1, as illustrated by the diagramed model (Figure 8). We show that ERK1 Y210 nitration, and probably phosphorylation as well [11], disrupts the critical H-bond between Y210 and E237, and results in misfolded ERK1. The latter recruits Hsp90 and its co-chaperon Cdc37 for the attempt of refolding. If the H-bond can be re-connected through the removal of the PTM on Y210, the refolding attempt could result in functional ERK1 with correct conformation. However, if the PTM cannot be removed quickly and the disruption of the H-bond is long-lasting as represented by the misfolded ERK1 species such as Y210F and E237A, the refolding attempt will fail. Subsequently, the quality control program will be initiated by recruiting HSP70 and CHIP for the ubiquitination and degradation of the aberrant ERK1. As such, the cytotoxicity from the aberrant accumulation and aggregation of misfolded ERK1 can be eliminated. This mechanism provides better flexibility for the quality control of ERK1. When ERK1 activity is not necessary under certain stress conditions, modification on Y210 inhibits ERK1 activity and nuclear localization. If the stress is long-lasting and modification on Y210 is stable, then the cytotoxic aberrant ERK1 can be scavenged through the quality control mechanism. If the stress is transient and the modification is subsequently removed after the stress, the misfolded ERK1 species could be recycled through refolding. Such flexibility allows cells to more effectively and economically regulate ERK1 activity.

The results from the site-specific mutations firmly establish that correct formation of the H-bond between Y210 and E237 is critical for optimal ERK1 activity and localization. Thus, disruption and formation of the H-bond, which is decided by the modification status of Y210, could act as a molecular switch for the quality control of ERK1. Here, we show that Y210 can be nitrated *in vitro* or under physiological conditions, and the nitration facilitates the breakage of the H-bond between Y210 and E237 as we recently showed using molecular dynamics simulation [29]. Protein nitration has been established as a biomarker for nitroxidative stress in cells and tissues, and has been implicated in diseases such as atherosclerosis and inflammation through regulating activities of the target proteins [30]. In addition, accumulating evidence has implicated protein nitration as a signaling event [31–33]. In human, ERK tyrosine nitration has been previously documented in different diseases such as portal hypertensive gastropathy [12] and could also serve as a signaling event by positively or negatively regulate ERK phosphorylation/activity [12,13], depending on which sites being nitrated. In rat, ERK tyrosine nitration was suggested to regulate Akt activity [34]. However, no specific site of ERK tyrosine nitration has previously been identified and how the nitration regulates ERKs activity has remain elusive. Our findings clearly demonstrate that Y210 nitration inhibits phosphorylation of the canonical ERK1 TEY motif (Figure 1A) and promotes ERK1 ubiquitination and HSP90 binding. Binding of HSP90 prevents ERK1 nuclear translocation and subsequently switches on the ERK1 degradation program, which in turn effectively turnover ERK1 when its activity is unnecessary. Such an interplay between nitration, phosphorylation, and ubiquitination represents an important mechanism to regulate signal transduction in cells [35,36].

In addition to the nitration identified in the current study, phosphorylation is the other potential PTM reported to occur on ERK1 Y210 [11]. Phosphorylation can also disrupt the H-bond between Y210 and E237 because the conjugated phosphate group on Y210 replaced the hydrogen necessary for the H-bond formation. If this is the case, Y210 phosphorylation could play a negative role in regulating ERK1 function, as also suggested by Lai and Pelech [11]. Interestingly, Lai and Pelech failed to identify the phosphorylation of Y210 by MS, though Lai and Pelech detected Y210 phosphorylation by Western blotting. In fact, no Y210 phosphorylation has been documented in any public phosphorylation databases. Lai and Pelech showed that the phosphorylation of Y210 was induced by MEK1 and followed the similar phosphorylation kinetics as that of the TEY motif, the canonical MEK substrate. Since modification on Y210 serves as a negative regulator of ERK1, as suggested by the findings in both Lai and Pelech and the current study, it would be interesting to ask how the positive and the negative signals, which are TEY phosphorylation and Y210 phosphorylation, respectively, are temporally co-ordinated to regulate ERK1 activity. Considering that both signals are simultaneously induced by activated MEK and follow the similar activation kinetics as suggested by Lai and Pelech [11], it would be a great challenge to co-ordinate such signals with opposing effects.

Binding of Hsp90 is a strong indicator of abnormal folding of Y210F. Hsp90 has been shown to indirectly regulate ERK activity positively or negatively in different scenarios [37,38]. Though ERKs are not previously considered as a *bona fide* client of Hsp90 [38], specific binding of Hsp90 to ERK1 was recently identified by quantitative MS [39]. Here, we show that HSP90 preferentially binds Y210F instead of ERK1 to prevent nuclear entry of the malfunctioning kinase, and binding of Hsp90 to ERK1 is substantially enhanced by MG132 treatment (Figure 3B), presumably due to accumulation of misfolded and malfunctioning ERK1 species that are otherwise targeted for degradation. Because numerous ERK substrates essential for gene transcription are in the nucleus, inhibition of nuclear entry of the abnormally folded ERK1 may serve as the other tier of quality control for the important kinase.

Previous evidence has established that CHIP is important in clearance of Hsp70/Hsp90 substrates such as the glucocorticoid receptor CFTR and the ErbB2 receptor [20,40]. Consistently, in the current study, CHIP was identified along with Hsp90, Cdc37, Hsp70, and Hsc70 as an interacting protein of Y210F. This clearly implicates its role in triage decision for the misfolded ERK1 resulting from Y210 mutation or modification, i.e. degradation through the ubiquitin–26S proteasome system. MEKK1 has previously been shown as the major E3 ubiquitin ligase for ERKs under osmotic stress [28]. Consistent with this, we also found that depletion of MEKK1 partially inhibits ubiquitination and degradation for both ERK1 and Y210F (Figure 5B–E,G). However, the inhibition is, to a much less extent, compared with the depletion of CHIP (Figure 5B–E,G). Thus, we conclude that CHIP, but not MEKK1, takes the major role to control Y210F ubiquitination and degradation under the current culturing condition. Particularly, CHIP preferentially controls the ubiquitination and degradation of Y210F over ERK1 (Figure 5C,E), and this highlights its significance and specificity in the quality control of ERK1 because Y210F represents a subpopulation of misfolded ERK1 species that can also be caused by PTM-induced disruption of the H-bond, a naturally occurring process. The preferential binding of Hsp90 to Y210F may dictate its degradation pathway, because chaperon association is probably a prerequisite for CHIP- but not MEKK1-mediated ERK1 ubiquitination and degradation, as previously demonstrated for CHIP-mediated CFTR ubiquitination [41]. Indirect regulation of ERK activity by CHIP through controlling the degradation of MEKK2 in response to hyperosmotic stress has previously been reported [42]. However, no previous evidence has shown that CHIP directly interacts with ERKs and controls its ubiquitination and degradation. Thus, our findings provide the first piece of evidence that CHIP directly regulates ERK1 activity.

The current study clearly demonstrates the importance of the H-bond between Y210 and E237 in regulating ERK1 conformation and activity. However, for ERK2, the H-bond is less important (Figure 7). ERK1 and ERK2 are highly similar in both amino acid sequence and functions. The activated ERK1 and ERK2 can phosphorylate the same set of substrates [2] and be regulated spatiotemporally by the same mechanism. In fact, the functions of the two isoforms have long been considered to be redundant until the evidence from ERK-knockout mouse showed that the two isoforms have different functions [43,44]. Recent evidence attributed the functional difference of the two isoforms to the difference of their expression level [45]. However, our findings strongly indicate that the activities and the quality control mechanism of the two isoforms are also differentially regulated (Figure 7), which are probably independent of their expression level.

## Abbreviations

CHIP: C-terminal of Hsp70-interacting protein
CHX: cycloheximide
ERK: extracellular-regulated protein kinase
FBS: fetal bovine serum
HRP: horseradish peroxidase
Hsp90: heat shock protein 90
IPed: immunoprecipitated
LMB: leptomycin B
MEK: mitogen-activated protein kinase kinase
MEKK1: mitogen-activated protein kinase kinase kinase 1
MS: mass spectrometry
NES: nuclear export signal
NTS: nuclear translocation signals
PDB: Protein Data Bank
PTM: post-translational modification
SPS: Ser-Pro-Ser
WCL: whole cell lysates
