# Supplementary material for: Nitration-induced ubiquitination and degradation control quality of ERK1
Source: Biochem J. 2019 Jul 2;476(13):1911–26. doi: 10.1042/BCJ20190240 (PMC6604951; doi:10.1042/BCJ20190240)
Supplement: Supplementary Figures and Tables [file BCJ-476-1911-s2.pdf]

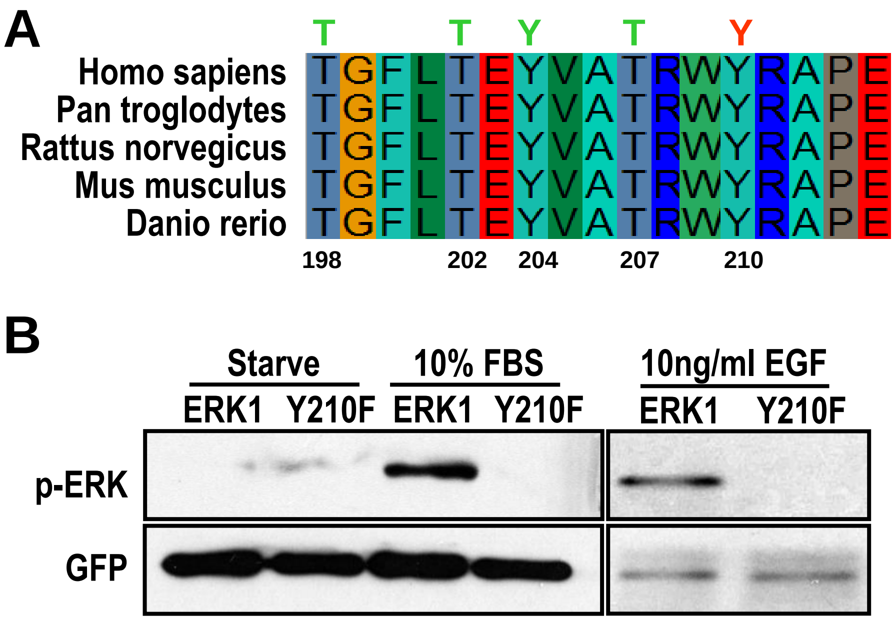

Figure S1 Site-directed mutagenesis of potential ERK1 phosphorylation sites. (A) Alignment of ERK1 sequences from different organisms shows the conservativeness of the known and potential phosphosites. (B) 293T expressing GFP fusion of ERK1 and its site-specific mutant Y210F were serum-starved overnight and then stimulated with 10% FBS or 10 ng/ml EGF for 10 minutes. Whole cell lysates from treated cells were assayed for ERK1 phosphorylation (T202/Y204) with Western-blotting using phospho-ERK antibody. Total ectopically expressed GFP fusion proteins were also detected as the loading control.

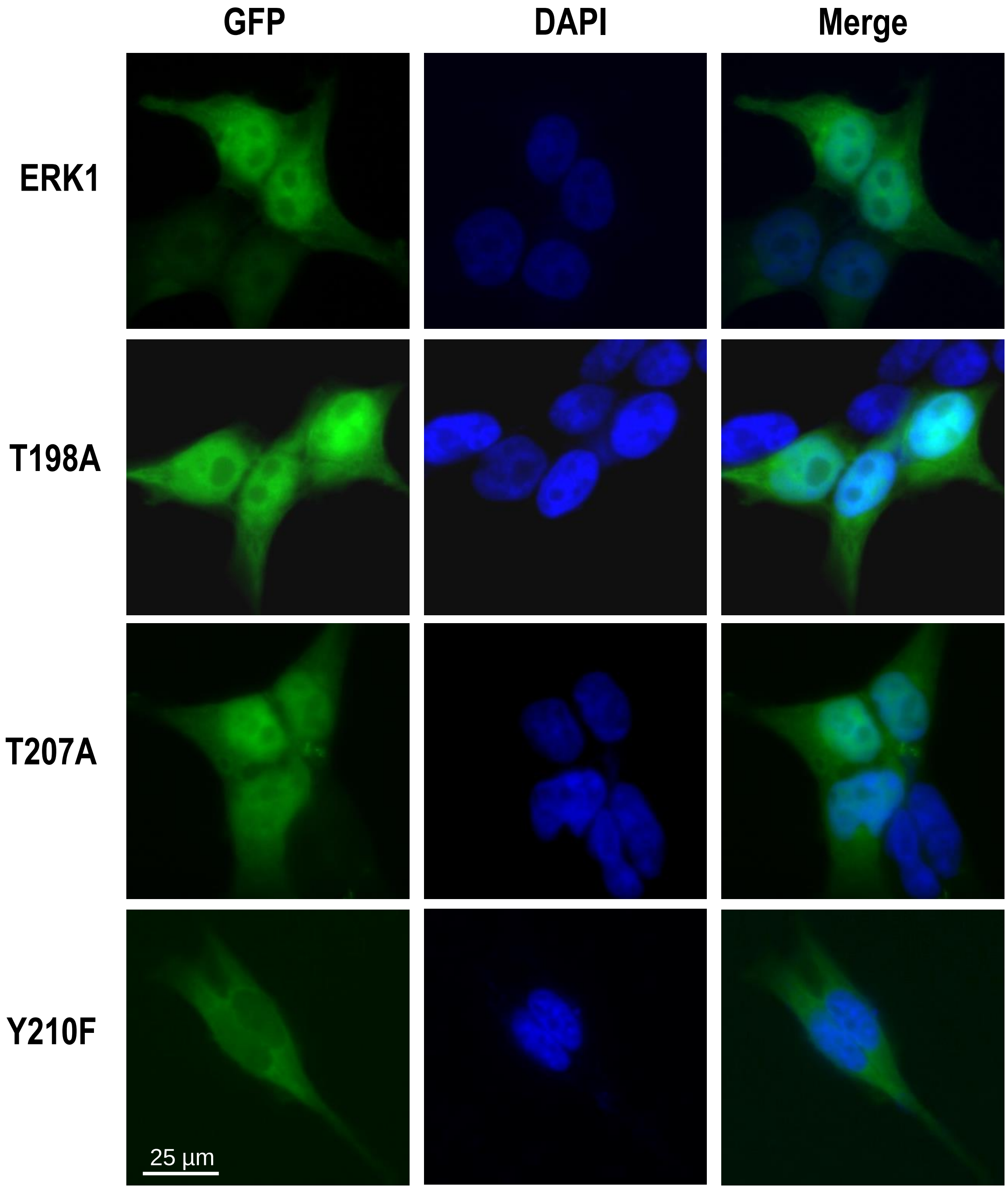

Figure S2. Nuclear-localization analysis of ERK1 site-specific mutants. 293T cells expressing GFP fusion of ERK1 and its site-specific mutants as indicated were stained with DAPI, and imaged with fluorescence microscopy.

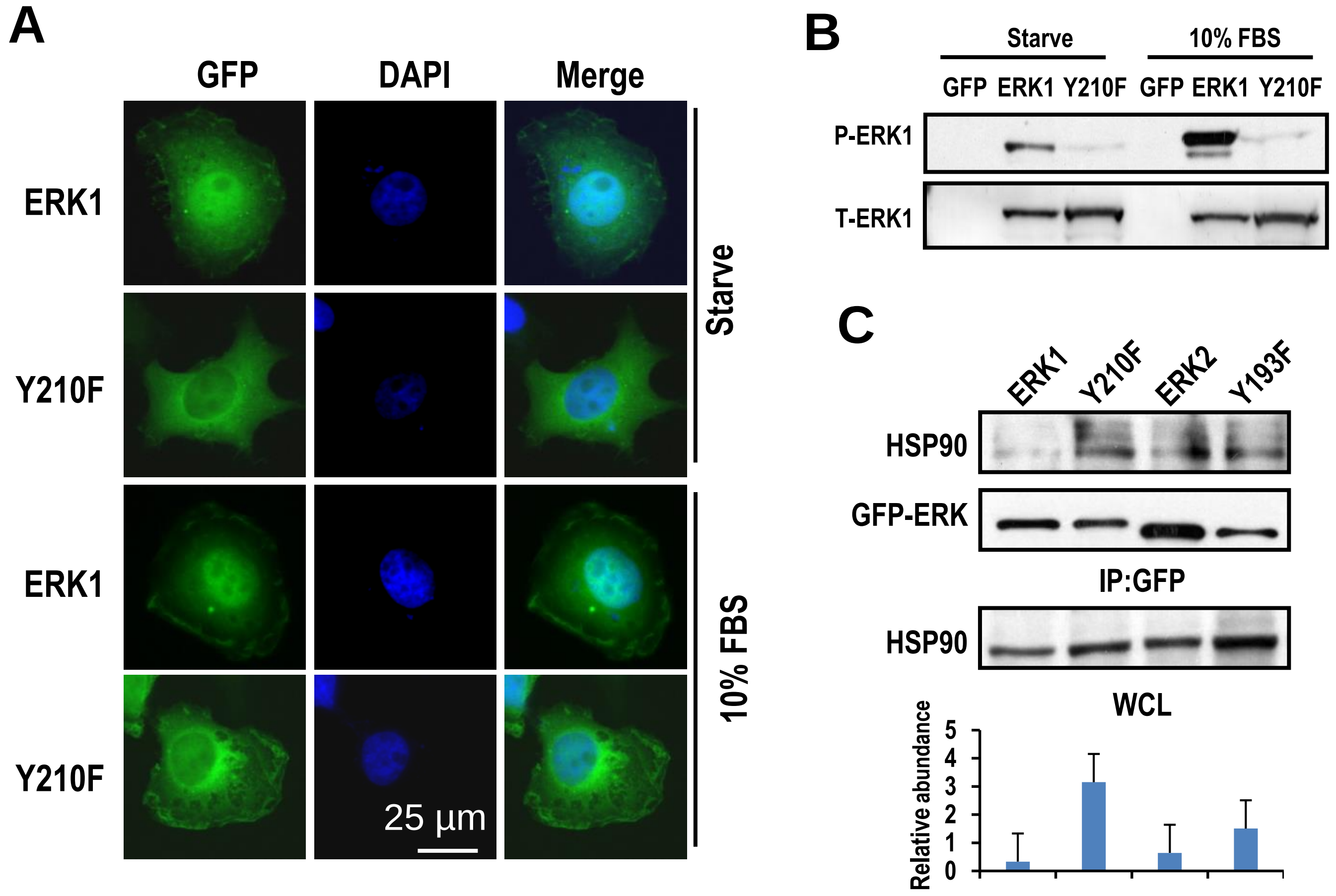

Figure S3. Y210F mutation abrogates ERK1 nuclear localization in HeLa cells. (A) HeLa cells expressing GFP-ERK1 (ERK1) or GFP-Y210F (210F) were stained with DAPI, and imaged with fluorescence microscopy. (B) Serum-starved HeLa cells expressing GFP-ERK1 or GFP-Y210F were stimulated with 10% FBS for 10 min or untreated (Starve). The cells were then lysed and probed for TEY phosphorylation of ERK1 or Y210F by Western-blotting (P-ERK1). Total levels of fusion proteins were also probed as the loading control (T-ERK1). (C) GFP-fusions of indicated proteins or their point mutants were IPed from the corresponding transfected Hela cells, and co-IPed Hsp90 was probed by Western-blotting. Total Hsp90 in the transfected cells was also probed as the loading control.

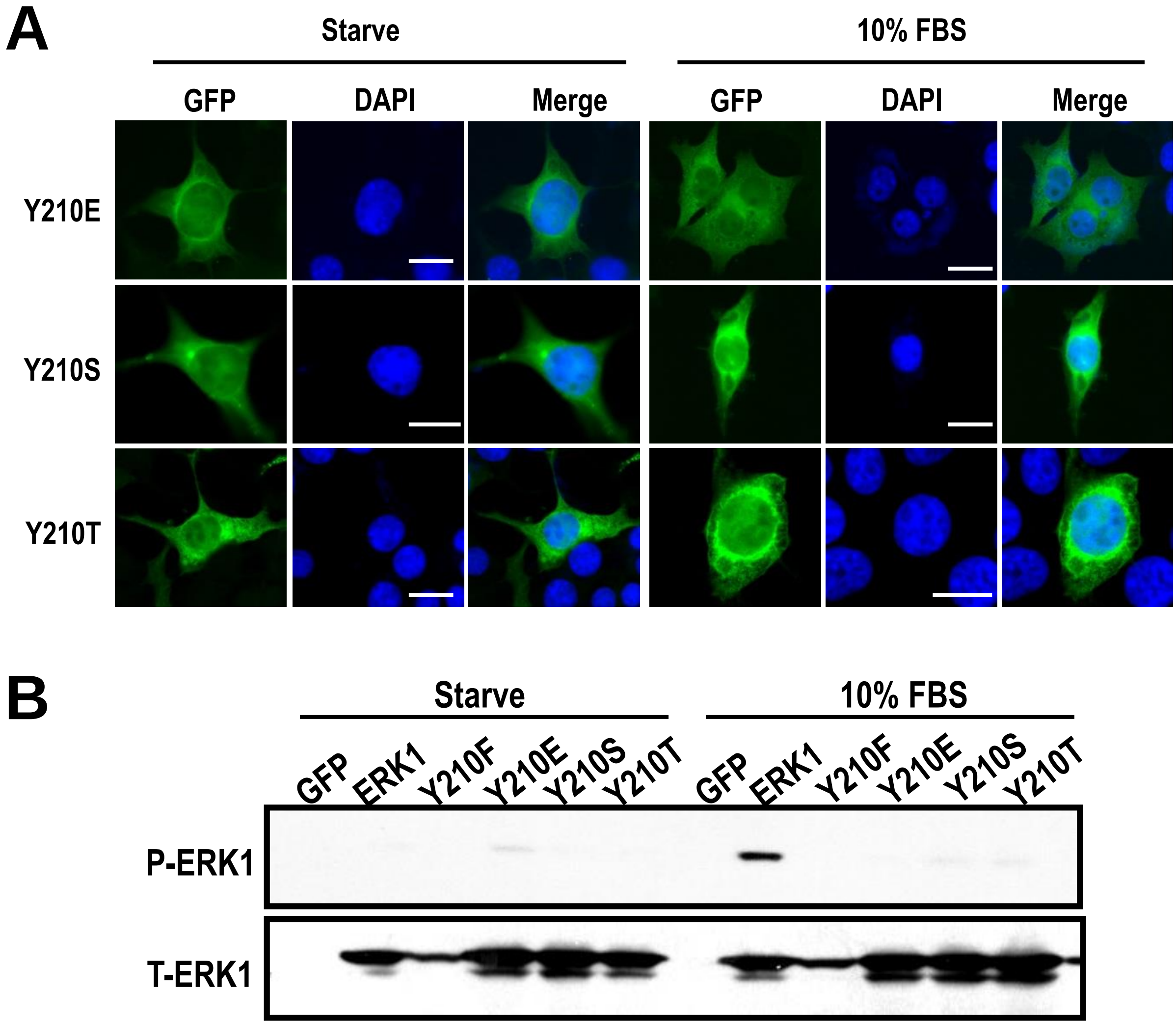

Figure S4. Y210 is critical for ERK1 phosphorylation and its nuclear localization. (A) 293T cells expressing ERK1 (GFP-ERK1) or its site-specific mutants Y210E, Y210S, and Y210T (GFP-Y210E, GFP-Y210S and GFP-Y210T) were serum-starved overnight, and then stimulated with 10% FBS for 10 min or unstimulated (Starve). The cells were then fixed, stained with DAPI, and imaged with fluorescence microscopy. Bar = 25 μm.

(B) The levels of total (T-ERK1) or phosphorylated (P-ERK1) GFP fusion proteins in cells same treated in A were detected by Western-blotting. Cells expressing GFP were also used as a control.

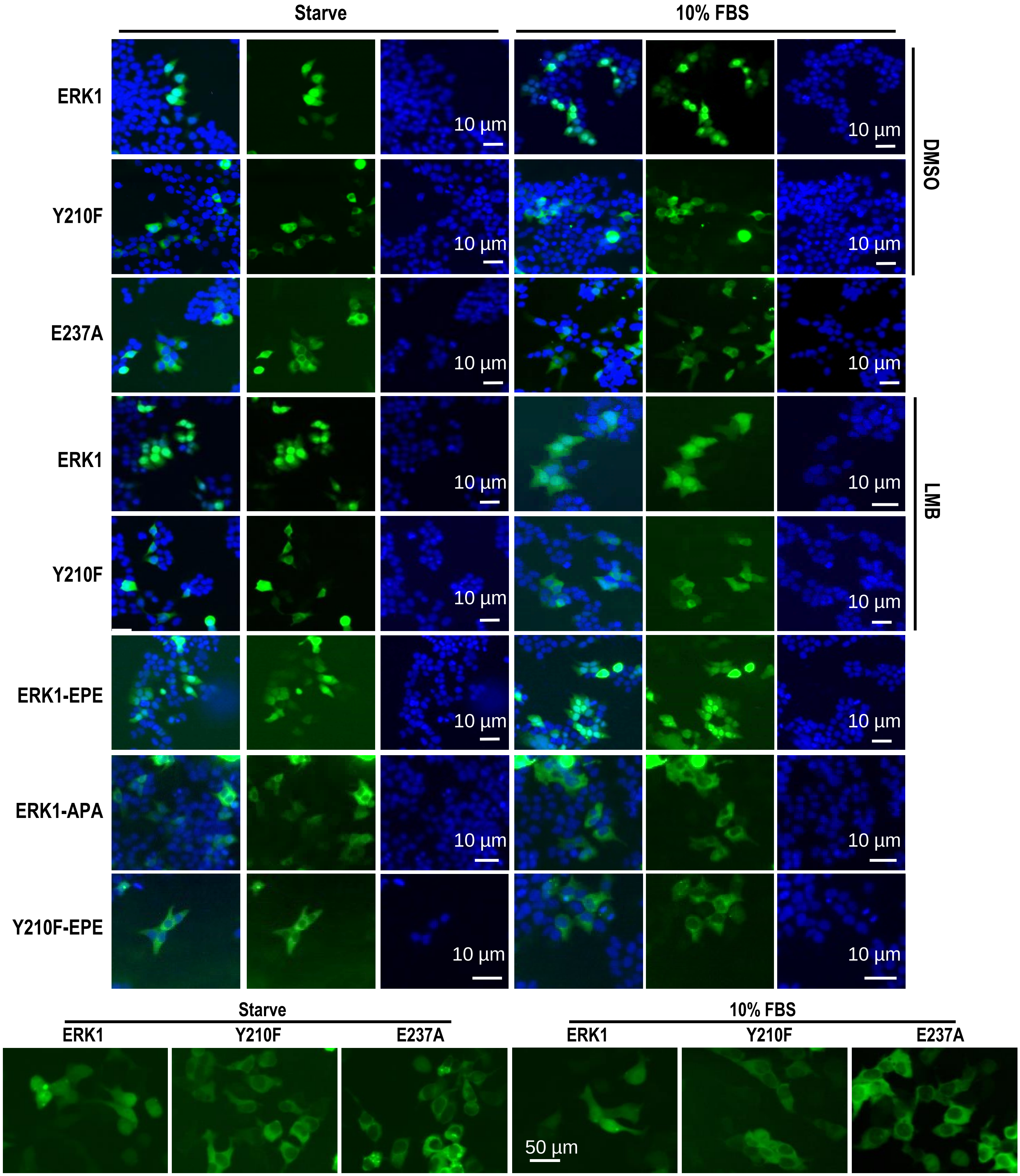

Figure S5. Y210 is critical for ERK1 nuclear localization. HEK293T cells expressing ERK1 (GFP-ERK1) or its site-specific mutants as indicated were serum-starved overnight, and then stimulated with nuclear export inhibitor LMB (40 mM) or vehicle (DMSO) for 3 hours as indicated. The Cells were then stimulated with 10% FBS or unstimulated (Starve) for 10 minutes as indicated. The cells were then fixed, stained with or without DAPI, and imaged with fluorescence microscopy.

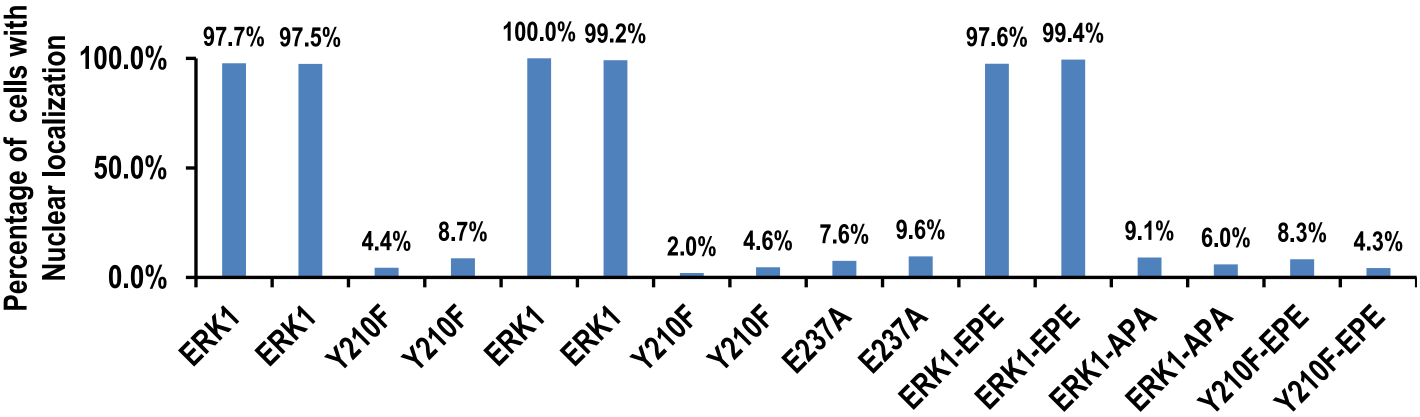

10% FBS	-	+	-	+	-	+	-	+	-	+	-	+	-	+	-	+
LMB					+	+	+	+								
Fields	17	13	17	10	11	11	10	10	19	14	10	10	12	12	10	10
Cell numbers	399	276	749	343	147	122	202	216	553	312	287	181	296	234	205	280

Figure S6. The bar graph shows the percentage of the cells with nuclear-localized GFP-ERK1 or GFP fusions of ERK1 site-specific mutants shown in Figure S5. The results were quantified from more than three different experiments by Image J.

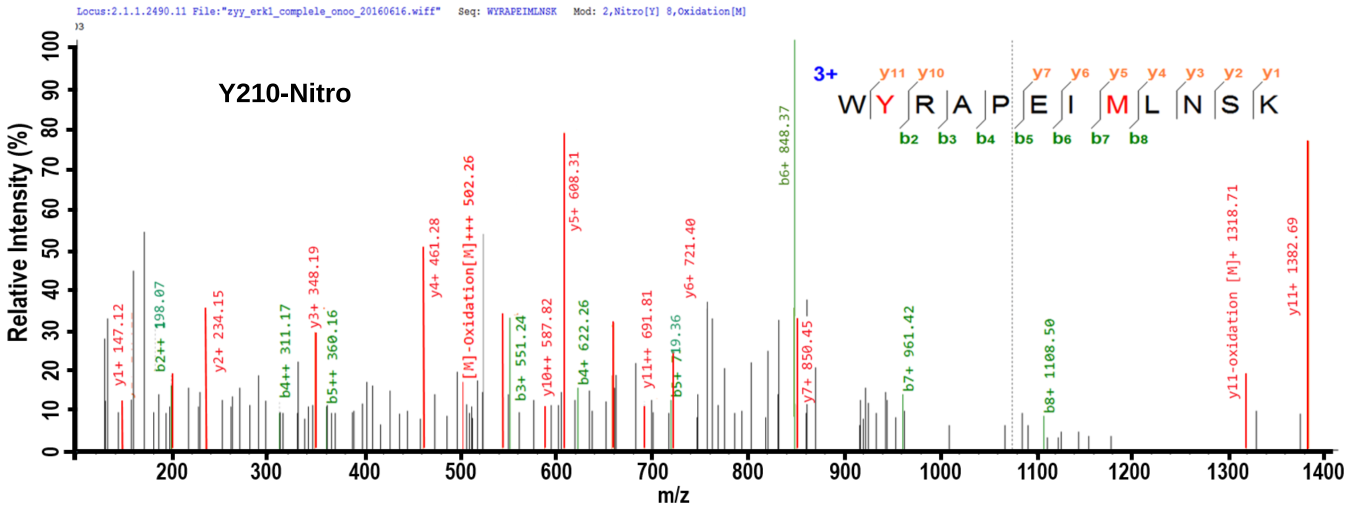

Peptide	PTM sites	score	Calculation Mass (Da)	Delta Mass (Da)	Delta Mass (ppm)
WyRAPEImLNSK	2, Nitro[Y];8, Oxidation[M];	1.53E-14	1567.745	-0.0043	-2.74104

Figure S7. Identification of nitro-Y210 from in-vitro nitrated ERK1. In vitro tyrosine nitration assay of ERK1, GST-ERK1 was pulled down from WCL, and then incubated with freshly collected WCL of 293T cells in the presence of 500 mM peroxynitrite. The fusion protein was then separated with SDS-PAGE and identified by MS. The table include the details about the mass accuracy of the MS analysis for nitro-Y210 peptide. The search was performed against Swiss-prot human proteome database using pFind. Score < E-03 is usually considered as a confident identification.

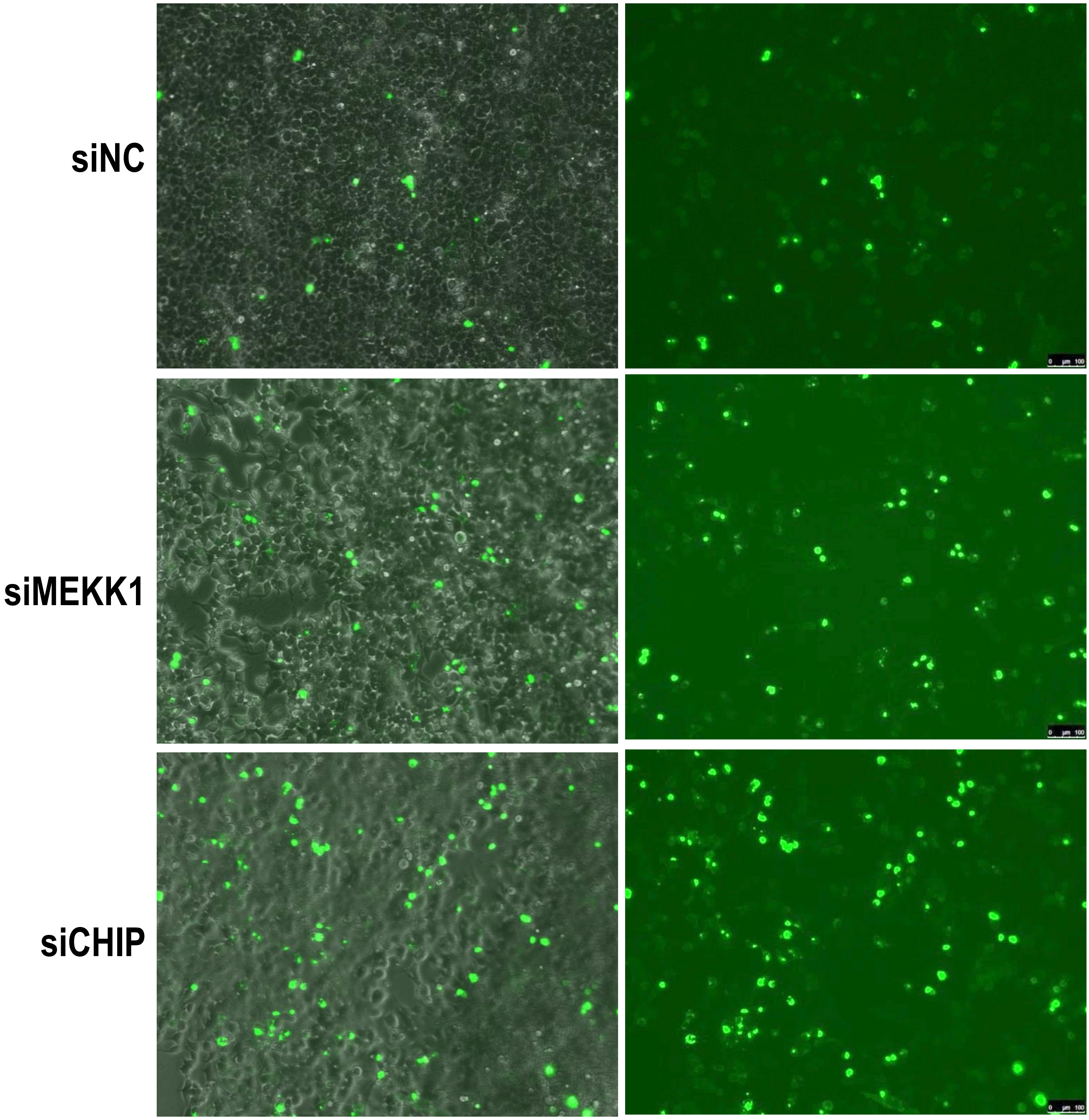

Figure S8. CHIP is the major E3 ubiquitin ligase mediating Y210F aggregation. 293T cells expressing GFP-Y210F were transfected with siRNA targeting for MEKK1 (siMEKK1) or CHIP (siCHIP) or non-targeting siRNA (siCtrl) as indicated. The cells were imaged with fluorescence microscopy. Phase contrast images were also shown to indicate the population of the cells.

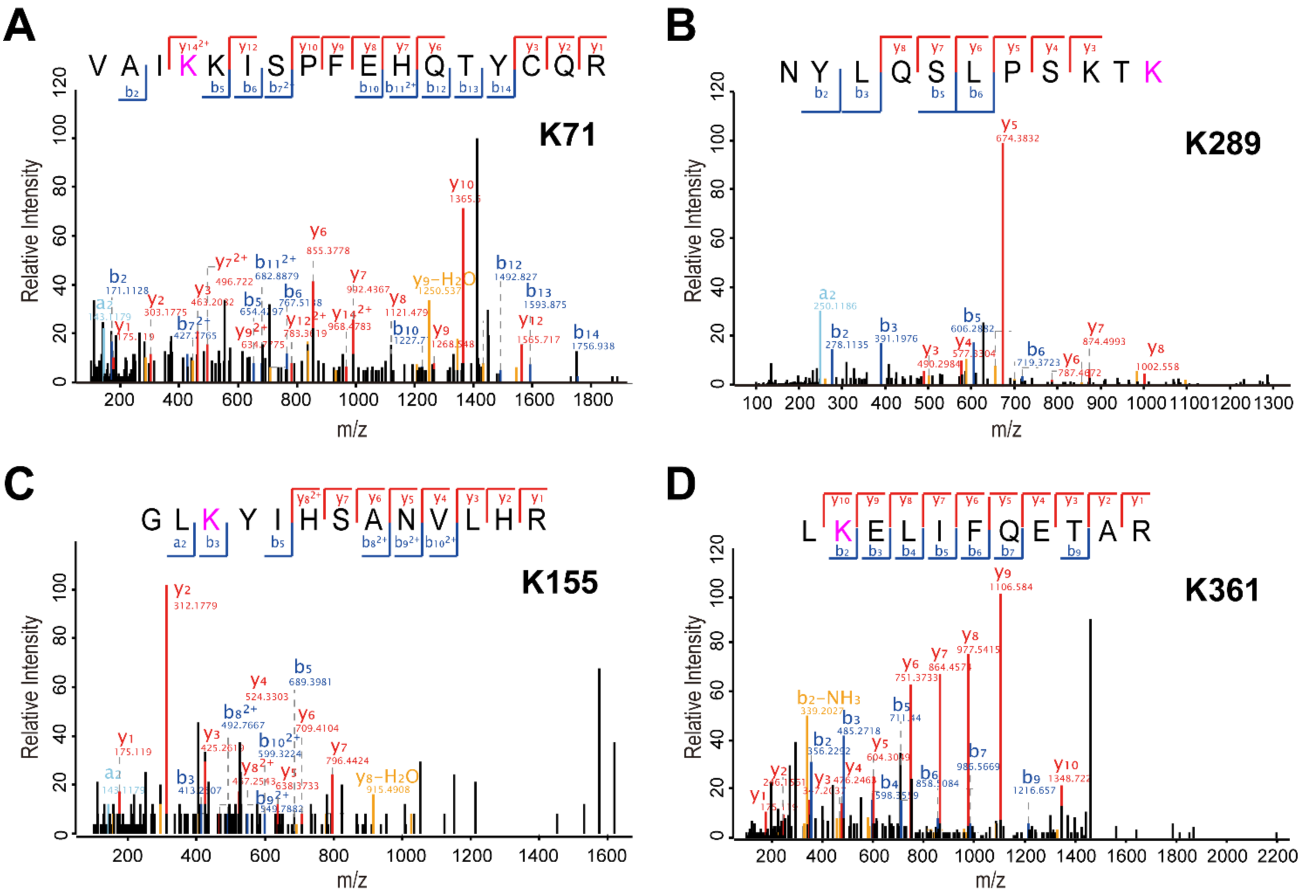

Figure S9. Identification of Y210F ubiquitination sites. (A-D).The mass spectra of peptides containing ubiquitinated K71, K289, K155, or K361 from Y210F. The ubiquitinated lysine residue in each peptide sequence is indicated in pink.

Supplemental Information

Immunoprecipitation and Western blotting

For immunoprecipitations, cells were lysed in RM buffer (1% NP-40, 0.1% deoxycholate, 150 mM NaCl, 1mM EDTA, 50 mM Tris, pH 7.5, 1 mM Na orthovanadate, 10 mM β-Glycerolphosphate, 10 mM sodium fluoride and Protease inhibitors). The lysates were precleared by incubation with protein A-agarose beads coupled with secondary mouse-anti-goat antibody for 2 hours， and the precleared lysates were then incubated with the indicated primary antibodies for 12 hours at 4 °C. After incubation, the immunoprecipitation complexes were washed with RM buffer to remove non-specific bound proteins, and the specific bound proteins were released from the beads by boiling with sample buffer (60mM Tris-Cl pH 6.8, 2% SDS, 10% glycerol, 5% beta-mercaptoethanol, 0.01% bromophenol blue). The immunoprecipitated proteins were then separated by SDS-PAGE and subjected to Western blotting using appropriate antibodies.

Immunofluorescence

Transfected 293T cells were plated on coverslips with approximately 50-60% confluence and allowed to attach and spread overnight in the starvation medium. The cells were then either treated with 10% FBS for 10 min to activate ERKs or untreated. After the treatment, the cells were washed with ice-cold

PBS, and fixed with 4% paraformaldehyde for 15 min, permeabilized with 0.1% Triton X-100 for 10 min, and then blocked with 10% BSA in PBS containing DAPI in a closed chamber at 37 °C for 1 h. The coverslips were subsequently washed, mounted on glass slides using anti-fade reagent, and visualized by Leica fluorescence microscopy (DMI6000B, Germany).

In-gel protein digestion and in-solution protein digestion.

For in-gel digestion of silver stained proteins, the gel containing the sample was cut into 2-3mm^2 pieces and washed in a buffer containing 3% H_2O_2 in 25mM ammonium bicarbonate to remove the staining [1]. In-gel digestion was subsequently performed according to a previously described procedure [2, 3]. Briefly, The destained gel slices were incubated with 10 mM DTT in 50 mM ammonium bicarbonate at 56 ℃ for 1 h and 55 mM iodoacetamide in 50 mM ammonium bicarbonate in dark for 45 min, and digested with trypsin (Promega, madison, WI; enzyme-to-substrate ratio 1:50) in 25 mM ammonium bicarbonate at 37℃ overnight. Peptides were extracted from gel by sonication in buffers containing 5% trifluoroacetic acid in 50% acetonitrile. The liquid was freeze dried by SpeedVac, and peptides were resolubilised in 10 μL 0.1% formic acid and filtered by 0.45μm centrifugal filter.

For in-solution digestion of IPed proteins, the beads bound with target proteins were resuspended with a denaturing buffer (8 M urea, 50 mM NH_4HCO_3, 1 mM sodium orthovanadate, 1 mM sodium fluoride,1/4 tablet

protease inhibitor cocktail per 10 ml). Tryptic digestion was performed using a well-established protocol [4]. Briefly, the proteins were reduced with 10 mM DTT at 37℃ for 1h and alkylated with 55 mM iodoacetamide in dark for 45 min. For the analysis of tyrosine nitration, the reduction and alkylation steps were omitted. The samples were further diluted with 50mM NH_4HCO_3 to reduce urea concentration from 8M to less than 2M before digestion. Protein digestion was performed with 5 ng/μL trypsin (Promega，madison, WI) at 37℃ overnight. The resulting tryptic peptides were desalted using C18 StageTips [5], and freeze-dried with a SpeedVac.

Mass spectrometry

The peptides were resuspended in 0.1% formic acid and analyzed by a TripleTOF 5600 mass spectrometer (AB SCIEX) coupled online to an Eksigent nanoLC Ultra in information-dependent acquisition mode. The parameters for LC-MS were set as previously described [6, 7]. Briefly, The LC gradient (A =0.1% formic acid in H_2O, B = 0.1% formic acid in acetonitrile) was 5–90% B for 90 min with a flow rate at 300 nL/min. MS spectra were acquired across the mass range of 350–1500 m/z in high resolution mode (>30,000) using 250 ms accumulation time per spectrum. A maximum of 25 precursors per cycle were chosen for fragmentation from each MS scan with minimum accumulation time of 2 s for each precursor and dynamic exclusion for 18 sec. Tandem mass spectra were recorded in high sensitivity mode (resolution >15,000) with rolling

collision energy on.

Data analysis

The database search was performed in the MaxQuant environment (version 1.5.3.28) or pFind when necessary [8-10]. For MaxQuant analysis, MS/MS spectra were searched using Andromeda against the decoy UniProt human proteome database, which contains forward and reverse sequences and is concatenated with 262 frequently observed contaminants including human keratins, bovine serum proteins, and proteases. The mass tolerances for the database search were set at 0.01 Da and 0.05 Da for precursor ions and fragment ions, respectively. Methionine oxidation and N-terminal acetylation were included as the variable modifications. For the identification of ubiquitination sites, diglycine resulting from tryptic digestion of conjugated ubiquitin was included as the variable modification [11]. Cysteine carbamidomethylation was included as the fixed modification. The maximal number of miscleavages was set at 2 and the minimum peptide length was set at 7 amino acids. The false discovery rates (FDR) for both peptide and protein identifications were set at 1%.

For the identification of tyrosine nitration, the database search was performed using the software pFind because high quality annotated spectra can be generated by this software for better visualization. Tyrosine nitration, methionine oxidation and N-terminal acetylation were included as the variable

modifications. Cysteine carbamidomethylation was included as the fixed modification. The maximal number of miscleavages was set at 4. The other parameters were automatically set to the default values.

References

1 Sumner, L. W., Wolf-Sumner, B., White, S. P. and Asirvatham, V. S. (2002) Silver stain removal using H2O2 for enhanced peptide mass mapping by matrix-assisted laser desorption/ionization time-of-flight mass spectrometry. *Rapid Commun Mass Spectrom*. **16**, 160-168 http://doi.org/10.1002/rcm.555

2 Wang, Y., Sun, J. and Chitnis, P. R. (2000) Proteomic study of the peripheral proteins from thylakoid membranes of the cyanobacterium Synechocystis sp. PCC 6803. *Electrophoresis*. **21**, 1746-1754 http://doi.org/10.1002/(SICI)1522-2683(20000501)21:9<1746::AID-ELPS1746>3.0.CO;2-O

3 Shevchenko, A., Tomas, H., Havlis, J., Olsen, J. V. and Mann, M. (2006) In-gel digestion for mass spectrometric characterization of proteins and proteomes. *Nature protocols*. **1**, 2856-2860 http://doi.org/10.1038/nprot.2006.468

4 Washburn, M. P., Wolters, D. and Yates, J. R., 3rd. (2001) Large-scale analysis of the yeast proteome by multidimensional protein identification technology. *Nat Biotechnol*. **19**, 242-247 http://doi.org/10.1038/85686

5 Wisniewski, J. R., Zougman, A. and Mann, M. (2009) Combination of FASP and StageTip-based fractionation allows in-depth analysis of the hippocampal membrane proteome. *J Proteome Res*. **8**, 5674-5678 http://doi.org/10.1021/pr900748n

6 Gao, L., Shen, C., Liao, L., Huang, X., Liu, K., Wang, W., Guo, L., Jin, W., Huang, F., Xu, W. and Wang, Y. (2014) Functional proteomic discovery of Slr0110 as a central regulator of carbohydrate metabolism in Synechocystis species PCC6803. *Mol Cell Proteomics*. **13**, 204-219 http://doi.org/10.1074/mcp.M113.033803

7 Gao, L., Ge, H., Huang, X., Liu, K., Zhang, Y., Xu, W. and Wang, Y. (2015) Systematically ranking the tightness of membrane association for peripheral membrane proteins (PMPs). *Mol Cell Proteomics*. **14**, 340-353 http://doi.org/10.1074/mcp.M114.044800

8 Cox, J. and Mann, M. (2008) MaxQuant enables high peptide identification rates, individualized p.p.b.-range mass accuracies and proteome-wide protein quantification. *Nat*

Biotechnol. **26**, 1367-1372 http://doi.org/10.1038/nbt.1511

9 Li, D., Fu, Y., Sun, R., Ling, C. X., Wei, Y., Zhou, H., Zeng, R., Yang, Q., He, S. and Gao, W. (2005) pFind: a novel database-searching software system for automated peptide and protein identification via tandem mass spectrometry. *Bioinformatics.* **21**, 3049-3050 http://doi.org/10.1093/bioinformatics/bti439

10 Wang, L. H., Li, D. Q., Fu, Y., Wang, H. P., Zhang, J. F., Yuan, Z. F., Sun, R. X., Zeng, R., He, S. M. and Gao, W. (2007) pFind 2.0: a software package for peptide and protein identification via tandem mass spectrometry. *Rapid Commun Mass Spectrom.* **21**, 2985-2991 http://doi.org/10.1002/rcm.3173

11 Xu, G., Paige, J. S. and Jaffrey, S. R. (2010) Global analysis of lysine ubiquitination by ubiquitin remnant immunoaffinity profiling. *Nat Biotechnol.* **28**, 868-873 http://doi.org/10.1038/nbt.1654
